# A comparison of perceptual anticipation in combat sports between experts and non-experts: A systematic review and meta-analysis

**DOI:** 10.3389/fpsyg.2022.961960

**Published:** 2022-10-28

**Authors:** Zhen Zhang, Alessandro Piras, Chao Chen, Bin Kong, Dexin Wang

**Affiliations:** ^1^School of Elite Sport, Shanghai University of Sport, Shanghai, China; ^2^Key Lab of Human Performance, Shanghai University of Sport, Shanghai, China; ^3^Department of Biomedical and Neuromotor Sciences, University of Bologna, Bologna, Italy; ^4^Department of Physical Education, Fudan University, Shanghai, China

**Keywords:** expertise, visual search, eye movements, reaction time, response accuracy

## Abstract

In order to systematically evaluate perceptual anticipation between experts and non-experts for different kinds of combat sports, we needed to perform a comprehensive assessment. In this systematic review and meta-analysis, we searched four English-language and three Chinese-language databases that used expert/non-expert research paradigms, to explore perceptual anticipation in combat sports. We employed a random effects model for pooled analyses using the inverse variance method. We included 27 eligible studies involving 233 datasets in this meta-analysis. We observed large effect sizes for the differences between experts and non-experts in both response accuracy (1.51; 95% CI: 1.15 to 1.87, *p* < 0.05) and reaction time (-0.91; 95% CI: −1.08 to−0.73, *p* < 0.05). We also observed substantial differences between experts and non-experts in the mean duration of visual fixations per trial (1.51; 95% CI: −2.40 to −0.63, *p* < 0.05), but not in the visual fixation duration (0.16; −061 to 0.92, *p* = 0.69). Taken together, high-level combat athletes have more advantages in perceptual anticipation than lower-level athletes, showing faster and more accurate responses when facing the opponent's attacks, as well as focusing on fewer points of visual fixations than novice athletes. Different types of combat sports and stimulus presentations affect perceptual anticipation abilities to varying extents in relation to outcome measures, with more pronounced expertise in a stimulus that is closer to real-world situations.

**Systematic review registration:**

https://www.crd.york.ac.uk/prospero/display_record.php?ID=CRD42021226343, PROSPERO CRD42021226343.

## Introduction

Perceptual anticipation plays a key role in competitive sports (Ottoboni et al., 2014; van Maarseveen et al., 2018), especially in combat sports, where athletes cannot usually wait until an attack is performed and should instead ideally anticipate the upcoming attack based on prior cues or information available from the opponent's movements (De Quel and Bennett, 2016). Precise, accurate, and fast anticipation abilities form the premise for athletes to successfully execute techniques and strategies, and are among the key factors directly affecting the outcome of a competition (Krabben et al., 2019; Martínez et al., 2019). In combat sports (e.g., boxing, judo, fencing, taekwondo), athletes compete at close range and need excellent perceptual skills to anticipate, react and respond to the opponent's attacks (Ripoll et al., 1995; Piras et al., 2014; Allerdissen et al., 2017; Ma, 2017a). Athletes need to pay attention to their opponents and adjust their attack and defense strategies to the opponent's actions. Generally, it is of great significance for combat athletes to quickly identify useful details from a large amount of dynamically shifting information about the competition in order to predict the opponent's attacks and to react and respond quickly and accurately. This information could be derived from multiple sources with varied forms, such as opponent movement and posture, distance/fighting measure and even from the status of the match. Information could also be derived from kinematics and projectile motion. Such information could be recognized and processed by athletes through various forms, such as visual and auditory, to help them predict their opponents' behavior and make judgments that are more conducive for winning the game. Several studies have reported the difference in the identification and processing of information between professional and non-professional athletes. Evidence from event-related potentials (ERP) showed that expert taekwondo athletes had a more rapid and efficient visual search strategy with dynamic threat stimuli (Wu et al., 2017). Specifically, the ERP showed that the experts evoked greater P1 latency and amplitude and N1 amplitude during the early visual processing stages. Another study suggested that the excellent performance in elite fencers was the result of their mental advantage ability in the identification and decision-making during complex situation, and more effective adaptation in changing situation (Fu, 2010).

A common theme in perceptual anticipation is that experts use anticipation to overcome the limits of the reaction time. The ability of expert performers to exploit perceptual cues can lessen the temporal constraints required in a reaction time task (Buckolz et al., 1988). Experienced athletes could quickly focus on the most informative or ‘information rich' areas through visual search. Information rich areas are determined by a number of methods including the use of eye-tracking technology to examine point-of-gaze of experts compared to their less-skilled counterparts. In some experimental studies, the combination between spatial and temporal occlusions, or the mere exposure to visual stimuli, increase the capacity of the perceptual anticipation through training. The efficiency of perceptual training is usually demonstrated by results indicating significant improvements in response times and/or accuracy (Poulter et al., 2005). Specifically, *Reaction time* is the duration of time between stimulus onset and initiation of movement response. Perceptual anticipation is used to optimize the timing of the response to improve the probability that the response is successful given the constraints of the responder (Dicks et al., 2010). Besides, the accuracy of reacting correctly in many experimental studies has also been widely studied. *Response accuracy* represents the percentage of trials in which an athlete's responses are adapted to situational constraints and task demands. Moreover, several indices of visual search data between experts and non-experts have been considered (Piras et al., 2014; Milazzo et al., 2015). These measures include the *mean number of fixations* and the *mean fixation duration*. Different experimental tasks were set to test the above variables. For example, combined with different stimuli (non-sport specific or sport-specific) and responses (non-specific button/key or sport movements), it is possible to test different types of reaction times, such as simple reaction time and/or choice reaction time (Mouelhi Guizani et al., 2006). Additionally, some studies have required participants to perform *in-situ* combat movements, while others have done so in front of a screen or receive auditory guidance (Rosalie and Müller, 2013; Piras et al., 2014; Allerdissen et al., 2017). Together, these studies demonstrate the advantages of experts over non-specialists in receiving and processing different sensory information. Besides, the specific stimulus/stimuli that athletes need to respond were not clarified in most of studies.

The expert/non-expert research paradigm is one of the most widely used models in the field of perceptual anticipation (Williams et al., 2002). This paradigm compares performance between experts and non-experts by establishing different tasks and scenarios to identify internal mechanisms underlying the experts' advanced motor skills in order to explore how these competencies are developed and to improve the performance of suboptimal athletes (Ripoll et al., 1995; Mori et al., 2002; Chan et al., 2011; Ottoboni et al., 2014; Milazzo et al., 2015; Allerdissen et al., 2017; Bianco et al., 2017). To our knowledge, there are three review articles (both narrative and systematic reviews) in combat sport. Óscar et al. conducted a narrative review to study reaction time, anticipation, visual search, and information pick-up, as well as its impact on sport performance between experts and non-experts (Martínez et al., 2019). Russo et al. performed a systematic review to study anticipation, decision-making, visual-spatial attention, and executive function by sorting them according to the research settings, such as realistic videos and pictures, *in-situ* situations, and general stimulations. They reported better performance in response to real and simulated stimulations in skilled athletes compared to their less skilled counterparts. However, the general paradigms (e.g., GO/NO-GO task, visual perception speed, and choice reaction time tasks) produced some controversial results (Russo and Ottoboni, 2019). Krabben et al. (2019) combined ecological psychology and dynamic systems—the ecological dynamics approach—to understand the behavior of two athletes in a one-on-one combat situation, defined as interpersonal synergy. Adopting a synergetic approach to combat sports is necessary to capture the richness of the behaviors emerging when two athletes are engaged in combative interactions. In addition to combat sports, the difference in perceptual prediction ability between experts and novices in other types of sports is partly responsible for the difference in their performance. Williams et al. found that high-level soccer players managed to use their knowledge of situational probabilities (i.e., expectations) to anticipate future events (Williams, 2000). Skilled players use their superior knowledge to control eye movement patterns necessary for seeking and picking up important sources of information more effectively than their less skilled counterparts. However, none of these studies quantitatively synthesized how specific indicators may affect sport performance between experts and non-experts. As suggested by Russo et al., a comprehensive analysis is needed to understand the results that have emerged from the literature (Russo and Ottoboni, 2019).

Meta-analysis is a useful statistical tool to quantitatively summarize various effect sizes through the principle of weighted synthesis. In addition, the heterogeneity between studies could be well explored by using the meta-analysis technique. Therefore, we aimed to perform a systematic, comprehensive assessment of the differences in perceptual anticipation between experts and non-experts, to quantitatively evaluate the extent of the differences, and to explore potential factors of perceptual anticipation associated with sport performance.

## Materials and methods

We performed a systematic literature review using four English-language databases (PubMed, Web of Science, EBSCO-PsychARTICLES, and EBSCO-SPORTDiscus) and three Chinese-language databases (CNKI, Wanfang Data, and CQVIP) with predefined search terms (Supplementary Table S1) based on the Preferred Reporting Items for Systematic Reviews and Meta-Analyses (PRISMA) guidelines (http://www.prisma-statement.org/). Two independent researchers screened the titles and abstracts of papers to potentially include them if they were published before December 31, 2021, and if they met the following criteria: (1) research on perceptual anticipation in combat sports with the expert/non-expert paradigm; (2) quantitative measurements of variables related to perceptual anticipation (e.g., reaction times, response accuracy, number and duration of fixations) using an experimental apparatus; and (3) full texts of the studies were available. We excluded abstracts from congress meetings or conference proceedings, study protocols, news outlets, commentaries, dissertations, theses, reviews, and case reports. The two independent researchers scrutinized the full texts of the included studies after the initial screening (researcher A identified 40 studies, while researcher B identified 44) to assess overall eligibility based on the inclusion and exclusion criteria. A third researcher was consulted when the two reviewers disagreed in their assessment of the study, and a total of 42 studies were finally included. As for eligible studies, data were extracted from the publication characteristics of the included papers, such as type of sport(s), type of stimulus presentation(s) (e.g., experimental conditions), definitions of expert/non-expert groups, number of participants, types of variables, and the mean and standard deviation of the variables. The Supplementary material outlines the characteristics of each included study.

For studies that reported more than two groups of participants (e.g., an expert group, an intermediate group, and a non-expert group), we only included the highest and lowest levels of participants in the main analysis and conducted a further analysis that encompassed intermediate participants. We applied a modified Methodological Index for Non-Randomized Studies (MINORS) to systematically assess each eligible study (Supplementary Table S2) (Slim et al., 2003). We first described the combined estimates of different variables within expert and non-expert groups. Then, we meta-analyzed the effect size (standardized mean difference, SMD; calculated by Hedges' g method) between the two groups to quantitatively measure between-group variance. For each outcome measure, we calculated the weighted mean effect size and 95% confidence interval (CI) around the mean to determine whether the effects were significantly different from zero. Based on Cohen's criteria, we deemed an *effect size* < *0.2* to be a small effect, *between 0.2 and 0.8* to be a medium effect, and > *0.8* to be a large effect (Rice and Harris, 2005). Rooted in the random effects meta-analysis model, we used the inverse variance method to estimate the pooled effect of different variables.

We determined variability between different datasets using heterogeneity tests with Higgins' I^2^ statistic. We explored the reasons for variations among eligible studies and examined whether the variance was affected by the type of sport and stimulus (static/dynamic/*in situ*) or by the subgroup analyzed. We considered a dynamic stimulus when participants were stimulated by video; a static stimulus occurred when photos were shown (such as an opponent's attack) to the participants. Furthermore, we defined a stimulus with real-life situations, in which participants had to react to an opponent, as an *in-situ* stimulus. We performed all statistical analyses using R (version 4.0.1) with the “meta” package to conduct the meta-analysis. For all statistical tests, we considered a two-tailed *P* < 0.05 to be statistically significant.

## Results

**Figure 1:**
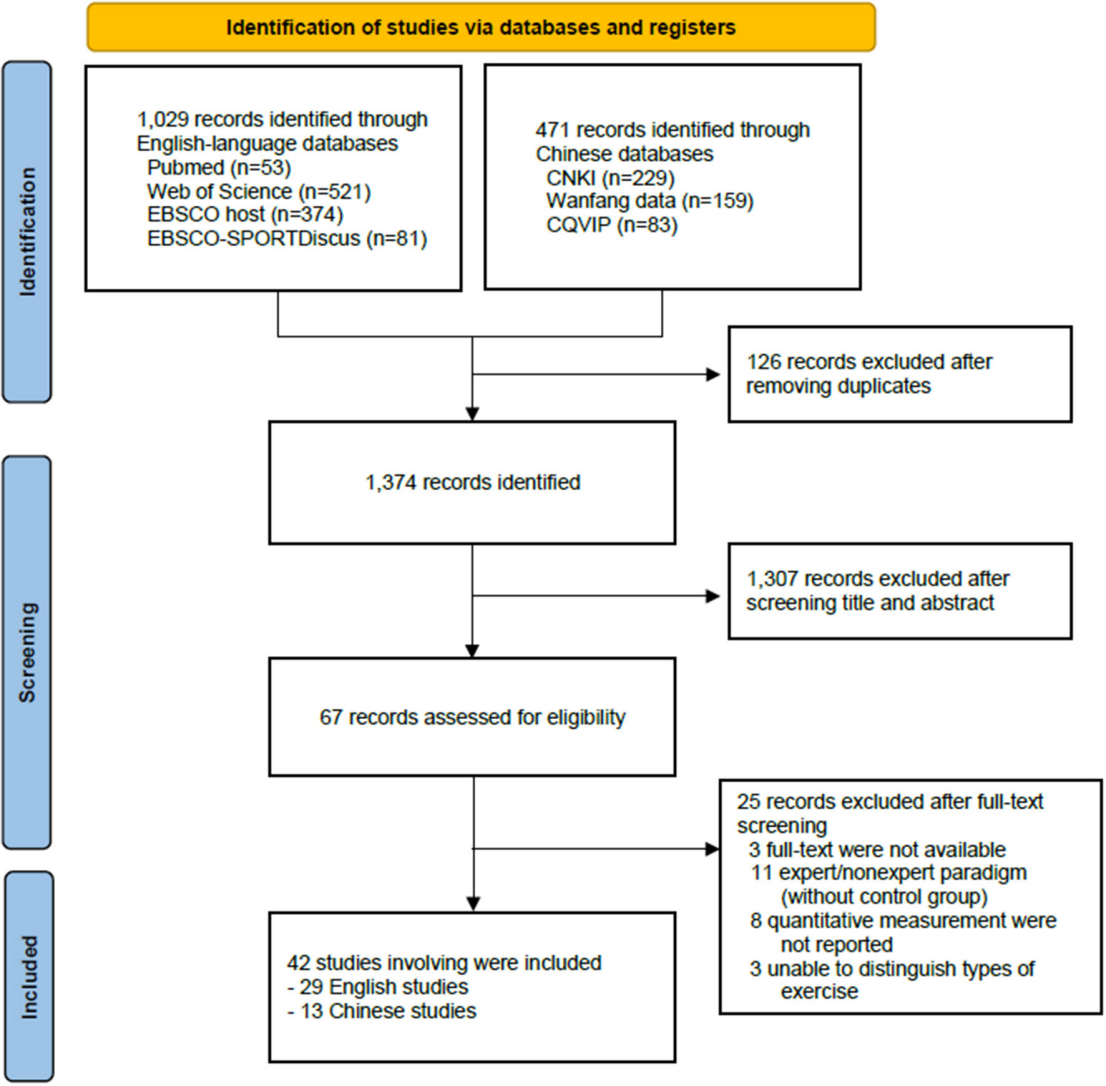
Flowchart of the selection of eligible studies.

After systematically searching for multiple data sources, we identified a total of 1,029 English-language studies and 471 studies. After removing duplicates and screening the titles and abstracts, we assessed 67 studies for eligibility. Finally, we included a total of 42 studies (including 29 English-language and 13 Chinese-language studies) reporting on perceptual anticipation in combat sports by using the expert/non-expert paradigm in the meta-analysis after completing full-text screening (Figure 1). We included all of the studies in our meta-analysis due to the generally good quality of research using the modified MINORS mentioned above (Supplementary Table S3).

Among the included studies, fencing (16 studies) and karate (10 studies) were the two types of sports examined with the highest frequency, followed by taekwondo (7 studies), boxing (6 studies), sanda (4 studies), and judo (3 studies). Regarding the determinants of perceptual anticipation, more than half of the studies measured response accuracy and reaction time in both experts and non-experts. Other indicators, such as the number of fixations and duration of fixations, were measured in a relatively small number of studies.

**Figure 2:**
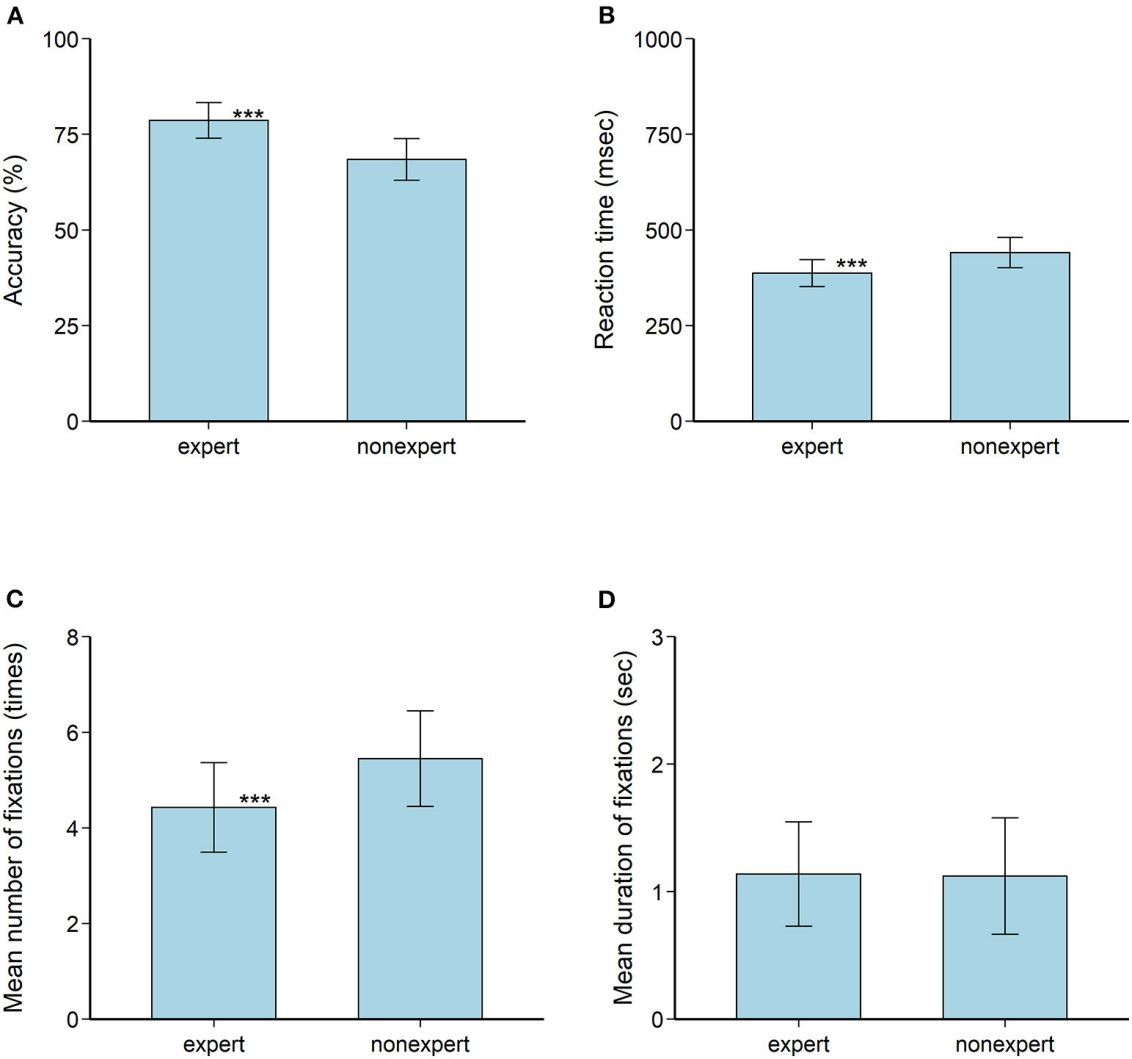
Pooled analysis of four indicators associated with perceptual anticipation among the expert and non-expert groups. **(A)** Accuracy. **(B)** Reaction time. **(C)** Mean number of fixations. **(D)** Mean duration of fixations. The vertical line represents the 95% confidence interval. The asterisks denote significant differences between the expert and non-expert groups for certain indicators.

Overall, the heterogeneity between studies was high, with a lower Higgins I^2^ of 0.77 for reaction time, compared to the other three major variables, with an even higher Higgins I^2^ above 0.90. Specifically, the response accuracy was higher in the expert group (83.3%) than in the non-expert group (68.5%) (Figure 2). The effect size for the difference between the two groups was a large effect of 1.24 (95% CI 0.80 to 1.68) according to Cohen's criteria, indicating that the probability of making the right decision was substantially higher in the expert than in the non-expert group when facing an opponent's attacks (Supplementary Figure S1, Table 1). Conversely, the pooled SMD for reaction time was −1.00 (95% CI: −1.14 to −0.86), suggesting that the reaction time to complete the stimulus task was shorter among experts than among non-experts (*p* < 0.05) (Supplementary Figure S2, Table 1). We also observed large differences between the two groups in the mean number of fixations (−2.04; 95% CI: −3.32 to −0.77), indicating that experienced athletes used a visual search strategy with a lower number of fixations than novices (Table 1). We found that the duration of fixations was not significantly different between experts and non-experts, with a medium effect of 0.64 (95% CI: −0.25 to 1.54), indicating that the time spent on fixation may not significantly different based on these pooled experimental data (Table 1).

**Table 1:** Pooled estimates of standardized mean differences between the expert and non-expert groups.

**Variable**	**No. of studies**	**SMD (95% CI)**	***P*** **value**	**I^2^ (P)**
Accuracy (%)	17	1.24 [0.80; 1.68]	*p < * 0.05	0.92 (*p < * 0.05)
Reaction time (ms)	34	−1.00 [−1.14; −0.86]	*p < * 0.05	0.77 (*p < * 0.05)
Mean number of fixations (times)	6	−2.04 [−3.32; −0.77]	*p < * 0.05	0.91 (*p < * 0.05)
Mean duration of fixations (sec)	6	0.64 [−0.25; 1.54]	*p =* 0.16	0.89 (*p < * 0.05)

Subgroup analyses further revealed that the performance level related to perceptual anticipation varied by sport and stimulus task. With the exception of fencers and boxers, the experts of other combat sports (e.g., karate, boxing, sanda, taekwondo) showed a higher accuracy than non-experts, with a range of SMD values from 0.14 to 3.08. Among the available data with reaction time, the experts reacted significantly faster than non-experts in most combat sports, including fencing (SMD: −1.07; 95% CI: −1.43 to −0.72), karate (-1.23; 95% CI: −1.47 to −1.00), judo (−0.54; 95% CI: −0.83 to −0.26), sanda (−1.36; 95% CI: −1.85 to −0.86), and taekwondo (−0.89; 95% CI: −1.12 to −0.66). In tasks involving either static or dynamic stimuli, experts performed more accurately and reacted faster than non-experts (*p* < 0.05), with the greatest differences during the *in-situ* stimulus, followed by dynamic and static stimuli (Table 2). Regarding other indicators, the type of sport and stimulus could partially explain the heterogeneity in the differences between the expert and non-expert groups (Table 2). Moreover, in fencing and judo, the mean number of fixations and the mean duration of fixations were greater among experts than among non-experts (*p* < 0.05) (Table 2).

**Table 2:** Subgroup analyses of standardized mean differences between the expert and non-expert groups by type of sport and stimulus presentation.

**Subgroup**	**No. of studies**	**SMD (95% CI)**	***P*** **value**	**I^2^ (P)**
Accuracy (%)				
Type of sport				
Fencing	6	0.14 [−0.42; 0.69]	*p* = 0.63	0.85 (*p < * 0.05)
Karate	5	3.08 [0.51; 5.65]	*p < * 0.05	0.90 (*p < * 0.05)
Boxing	2	0.52 [−0.17; 1.21]	*p =* 0.14	0.81 (*p < * 0.05)
Sanda	1	1.62 [0.85; 2.40]	*p < * 0.05	0.35 (*p* = 0.22)
Taekwondo	4	2.07 [1.51; 2.63]	*p < * 0.05	0.92 (*p < * 0.05)
Type of stimulus				
Static	9	0.57 [0.03; 1.10]	*p < * 0.05	0.91 (*p < * 0.05)
Dynamic	9	1.42 [0.88; 1.96]	*p < * 0.05	0.93 (*p < * 0.05)
In situ	1	7.71 [6.00; 9.43]	*p < * 0.05	0.00 (*p* = 0.40)
Reaction time (ms)				
Type of sport				
Fencing	14	−1.07 [−1.43; −0.72]	*p < * 0.05	0.75 (*p < * 0.05)
Karate	9	−1.23 [−1.47; −1.00]	*p < * 0.05	0.76 (*p < * 0.05)
Boxing	5	−0.51 [−1.04; 0.03]	*p* = 0.07	0.80 (*p < * 0.05)
Judo	2	−0.54 [−0.83; −0.26]	*p < * 0.05	0.63 (*p < * 0.05)
Sanda	2	−1.36 [−1.85; −0.86]	*p < * 0.05	0.41 (*p* = 0.15)
Taekwondo	6	−0.89 [−1.12; −0.66]	*p < * 0.05	0.78 (*p < * 0.05)
Type of stimulus				
Static	22	−1.00 [−1.20; −0.79]	*p < * 0.05	0.76 (*p < * 0.05)
Dynamic	13	−0.99 [−1.18; −0.81]	*p < * 0.05	0.77 (*p < * 0.05)
In situ	1	−2.18 [−3.14; −1.22]	*p < * 0.05	-
Mean number of fixations (times)				
Type of sport				
Fencing	2	−1.49 [−2.12; −0.87]	*p < * 0.05	0.00 (*p* = 0.87)
Karate	2	−3.20 [−8.39; 1.99]	*p* = 0.23	0.94 (*p < * 0.05)
Judo	1	−3.34 [−4.67; −2.00]	*p < * 0.05	0.74 (*p < * 0.05)
Sanda	1	−0.42 [−2.35; 1.51]	*p* = 0.67	0.94 (*p < * 0.05)
Type of stimulus				
Static	1	−0.42 [−2.35; 1.51]	*p =* 0.67	0.94 (*p < * 0.05)
Dynamic	3	−1.10 [−1.61; −0.59]	*p < * 0.05	0.14 (*p =* 0.32)
In situ	2	−4.21 [−6.14; −2.27]	*p < * 0.05	0.85 (*p < * 0.05)
Mean duration of fixations (sec)				
Type of sport				
Fencing	1	0.78 [0.01; 1.55]	*p < * 0.05	-
Karate	2	0.55 [−0.53; 1.63]	*p =* 0.32	0.76 (*p < * 0.05)
Judo	1	1.81 [0.37; 3.26]	*p < * 0.05	0.87 (*p < * 0.05)
Sanda	2	−0.29 [−1.93; 1.35]	*p =* 0.73	0.93 (*p < * 0.05)
Type of stimulus				
Static	1	−0.55 [−2.47; 1.37]	*p =* 0.58	0.94 (*p < * 0.05)
Dynamic	3	0.48 [−0.02; 0.98]	*p =* 0.06	0.68 (*p < * 0.05)
*In situ*	2	1.76 [0.60; 2.92]	*p < * 0.05	0.84 (*p < * 0.05)

We conducted further analysis for studies involving mid-level groups, and compared different indicators between expert/intermediate groups and intermediate/non-expert groups. Regarding accuracy rates, experts performed better than those at the intermediate level (SMD: 1.10; 95% CI: 0.63 to 1.58; *p* < 0.05), who in turn performed better than non-experts (1.37; 95% CI: 1.11 to 1.63; *p* < 0.05). We noted a similar tendency with reaction time (Table 3).

**Table 3:** Analyses of standardized mean differences between the expert, intermediate, and novice groups.

**Variable**	**No. of studies**	**Expert group vs. intermediate group**				**Intermediate group vs. novice group**		
		**SMD (95% CI)**	***P*** **value**	**I^**2**^ (P)**		**SMD (95% CI)**	***P*** **value**	**I^**2**^ (P)**
**Accuracy**								
Overall	7	1.10 [0.63; 1.58]	*p < * 0.05	0.85 (*p < * 0.05)		1.37 [1.11; 1.63]	*p < * 0.05	0.72 (*p < * 0.05)
Subgroup: Type of sport								
Fencing	2	0.29 [−0.33; 0.90]	*p =* 0.36	0.00 (*p =* 0.72)		−0.03 [-0.59; 0.53]	*p =* 0.93	0.00 (*p =* 0.47)
Karate	2	3.65 [0.71; 6.59]	*p < * 0.05	0.92 (*p < * 0.05)		2.96 [1.16; 4.76]	*p < * 0.05	0.85 (*p < * 0.05)
Boxing	1	−0.94 [−2.36; 0.47]	*p =* 0.19	0.86 (*p < * 0.05)		1.55 [0.57; 2.52]	*p < * 0.05	0.72 (*p < * 0.05)
Taekwondo	2	1.28 [1.02; 1.53]	*p < * 0.05	0.73 (*p < * 0.05)		1.36 [1.16; 1.57]	*p < * 0.05	0.58 (*p < * 0.05)
Subgroup: Type of stimulus								
Static	4	0.25 [−0.41; 0.91]	*p =* 0.46	0.86 (*p < * 0.05)		1.13 [0.79; 1.46]	*p < * 0.05	0.63 (*p < * 0.05)
Dynamic	4	1.58 [1.18; 1.98]	*p < * 0.05	0.79 (*p < * 0.05)		1.58 [1.19; 1.96]	*p < * 0.05	0.76 (*p < * 0.05)
Reaction time								
Overall	9	−0.34 [−0.49; −0.18]	*p < * 0.05	0.53 (*p < * 0.05)		−0.43 [-0.56;−0.31]	*p < * 0.05	0.37 (*p < * 0.05)
Subgroup: Type of sport								
Fencing	4	−0.37 [−0.63; −0.10]	*p < * 0.05	0.41 (*p < * 0.05)		−0.52 [-0.73;−0.31]	*p < * 0.05	0.29 (*p =* 0.12)
Karate	1	−1.12 [−1.89; −0.35]	*p < * 0.05	–		−0.09 [-0.82; 0.64]	*p =* 0.81	-
Boxing	1	−0.27 [−1.41; 0.87]	*p =* 0.64	–		0.50 [-0.66; 1.66]	*p =* 0.40	-
Taekwondo	3	−0.29 [−0.49; −0.10]	*p < * 0.05	0.63 (*p < * 0.05)		−0.41 [-0.57;−0.25]	*p < * 0.05	0.44 (*p < * 0.05)
Subgroup: Type of stimulus								
Static	6	−0.40 [−0.56; −0.24]	*p < * 0.05	0.26 (*p =* 0.11)		−0.47 [-0.65;−0.29]	*p < * 0.05	0.47 (*p < * 0.05)
Dynamic	4	−0.22 [−0.52; 0.07]	*p =* 0.14	0.73 (*p < * 0.05)		−0.40 [-0.56;−0.25]	*p < * 0.05	0.12 (*p =* 0.32)

## Discussion

To quantitatively evaluate the extent of the differences and to explore potential factors of perceptual anticipation associated with sport performance, we conducted a systematic, comprehensive literature search and meta-analysis to synthesize the available data. We found that the quality of the retrieved studies related to this topic was generally good. We also observed that professional combat athletes reacted faster and had greater accuracy in completing a target task (e.g., pressing a key or button, performing a certain movement in a sport, etc.), with higher efficiency than non-professional combat athletes. Different types of sports and stimuli published in the literature influence these differences to varying extents in relation to outcome measures.

Experts had higher accuracy in their task completion relative to their less skilled counterparts, with a large effect size observed. In addition, experts anticipated their opponents' intentions significantly earlier than non-experts. Response accuracy and reaction time are the two indicators that intuitively reflect predictive perception. These results are based on the idea that the use of advanced perceptual cues facilitates sport performance, supporting the anticipation of the opponent's intention and decreasing the overall reaction time (Shim et al., 2005). We noted large effect sizes of reaction time and response accuracy between experts and non-experts under both dynamic and static stimuli, as well as during *in-situ* situations, probably due to the longer training times of experts and their greater experience in competitions. Experts can store the experience and knowledge gained from trainings and competitions in long-term memory and can quickly extract this information when needed to swiftly and accurately execute an action (Ericsson and Chase, 1982). From an ecological perspective, skilled performers are better adapted to their environment and better attuned to early information, which enables them to employ their expertise *via* a stimulus that is closer to a real-world situation (*in situ* > dynamic > static) (Krabben et al., 2019). In contrast, non-expert athletes lack professional knowledge and game experience, and do not have an advantage in their speed of visual search and information processing; thus, they exhibit a lower accuracy and slower reaction times than their more skilled counterparts (De Quel and Bennett, 2016). These results are consistent with other sports such as badminton (Ye and Chi, 2010), table tennis, soccer, and basketball (Yin, 2013). The better use of advanced perceptual cues by skilled athletes has been confirmed to improve sport performance by accurately predicting the opponent's actions and accelerated reaction time.

In the course of a competition, athletes need to constantly be attuned to all kinds of relevant information, discard irrelevant information, and make accurate prejudgments and appropriate action responses in the shortest time possible. In the field of cognitive psychology, visual search strategy has become an important means to study cognitive characteristics (Wilschut et al., 2014; Mestry et al., 2017). The number of fixations and the corresponding durations are two frequently studied variables in perceptual anticipation in sports. We found that high-level combat athletes displayed a smaller number of fixations than their low-level peers. Meanwhile, the duration of fixations was not significantly different between the groups. This was an unexpected outcome, even though in fencing and judo—and most importantly, the *in-situ* experimental approach—we found that experts exhibited longer fixation durations in comparison to their non-expert counterparts. In prejudging the opponent's actions, high-level combat athletes showed a slightly lower number of fixation areas, with a more concentrated distribution in comparison to the scattered and irregular visual search strategy of non-experts (Ma, 2017b). High-level athletes have a more stable, deeper attention when predicting their opponent's attacks, with a visual search strategy structured in fewer fixations of longer duration (Babadi Aghakhanpour et al., 2021). Athletes have accumulated comprehensive, professional theoretical knowledge and experience, summarized in years of training and competitions, which have enabled them to selectively capture and preferentially process important information (Williams et al., 2002; Lin, 2014; Ma, 2017b). In contrast, low-level athletes have poor attentional stability and lack an in-depth understanding of specific techniques and essential movements. They used a visual search strategy with higher fixations of shorter duration, perhaps looking at their opponent's moving body segments, probably because they were unable to properly distribute their visuospatial attention. They might not have been trained to form a mature cognitive process, from using advanced cues and information, as well as receiving signals and stimuli, to making the right response. All of this results in a low efficiency of their visual search strategy, with unclear purpose of their intentional fixations (Yan, 2015; Witkowski et al., 2018, 2020).

Based on our findings, the type of stimulus material used may affect the differences between experts and non-experts for the various behavioral measures investigated. One potential explanation is that the spatiotemporal constraints of video-based (dynamic) tasks are higher than the photo-based (static) tasks (i.e., the dynamic tasks are more difficult); hence, it is easier to distinguish experts from non-experts. However, stimulus presentation modality is a critical moderating variable in the field of perceptual anticipation within expert/non-expert research paradigms (Mann et al., 2007). Learning with a dynamic, as opposed to static visual support, results in better learning outcomes among practitioners (Rekik et al., 2019). Our findings are consistent with previous studies, which affirmed that dynamic stimuli can provide more representative scenes that are in line with real sports situations in comparison to static stimuli (Mann et al., 2007). In addition, our outcomes indicate that the performance between experts and non-experts is most apparent when the tasks are set in a real-life situation (*in-situ*), suggesting that the closer the experimental situation is to the actual task, the better performance experts can accomplish with respect to non-experts.

However, other considerations must be taken into account, such as the sample size of the included studies, the representativeness of the task, time constraints, and perception-action coupling. More attention should be given to the limited data included in these analyses, and further studies are needed to compare the performance related to perceptual anticipation between experts and non-experts in light of different types of stimuli.

## Limitations

Our study has several limitations. First, the heterogeneity in the pooled analysis of potential factors—such as different combat sports, different experimental tasks, methods of measurements, the criteria and definitions used to group the participants, and non-standardized procedures associated with perceptual anticipation—could have influenced the results. We tried to use subgroup analyses to further examine the reasons underlying the variations, but the analysis was limited by insufficient datasets. Second, we did not consider other useful variables (e.g., decision-making) associated with perceptual anticipation due to the low number of articles involved. Finally, the definitions of “expert” and “non-expert” varied across the studies, which may have induced heterogeneity when pooling estimates from different studies. However, we conducted a sensitivity analysis to evaluate differences in perceptual anticipation at different levels of expertise.

## Future research

Future studies need to further specialize the type of stimulus/stimuli in their experimental task. Using simulation training, setting realistic situation and creating *in-situ* stimulus could give beginners more specific and helpful recommendation to improve their visual search behavior (Petri et al., 2017, 2020). Besides, future studies should focus on other variables associated with perceptual anticipation in sports, such as quiet eye and microsaccades. The former is supposed to be a period of time when task-relevant environmental cues are processed and motor strategies are synchronized for the successful completion of an upcoming task (Vickers, 1996). Precisely, the quiet eye period denotes the time spent between the last visual fixation on a target and the start of one's motor response (Vickers, 1996). The latter, the microsaccades, are small, rapid eye movements executed during fixations that can be used to detect the allocation of covert attention, revealing the link between visuomotor performance and covert attention shifts (Martinez-Conde et al., 2013). Microsaccades are important to anticipate the opponent's intention, modulated by visual attention and functionally related to saccadic intrusions. These microsaccades could improve the perception of the game, helping athletes during the period that precedes critical movement initiation, shifting from covert to overt attention, which is necessary to identify useful cues with both foveal and parafoveal vision (Piras et al., 2015, 2019, 2021a,b). The training of the perceptual-cognitive skill provides a good method for developing anticipation and decision-making in athletes, and research should assess whether improvements during acquisition could be transferred to the field. Practical implications for coaches and trainers should be encouraged to design tasks and give instructions to increase athletes' visual search behavior. Different studies have demonstrated that the way in which practice is planned influences the performance and learning skills (Broadbent et al., 2015).

## Practical implications

From a practical perspective, practitioners engaging athletes in simulation training to improve perceptual-cognitive skills should seek to promote high contextual interference to achieve long-term learning and transfer the skills acquired to the field. The contextual interference effect refers to the interference that is experienced when practicing multiple skills, or variations of a skill, within a single practice session (Intraub and Richardson, 1989). The effects of contextual interference on learning skills, for example, in combat sports (grip, lifting hand, pulling hand), are influenced by the kind of training session involved. This means that the activities can be carried out based on a repetitive practice schedule (blocked practice), presenting the same task continuously (low interference), or using a random practice schedule by performing more tasks (high interference). High contextual interference, even though it produces immediate limited performance, leads to superior performance in terms of retention and transfer tests in the field (Bortoli et al., 1992).

## Conclusions

In sum, high-level combat athletes have more advantages in perceptual anticipation than lower-level athletes, showing faster and more accurate responses, as well as focusing on a lower number of fixations than novice athletes. Different types of combat sports and stimulus presentations affect perceptual anticipation abilities to varying extents in terms of outcome measures, with a more pronounced expertise in a stimulus that is closer to real-world situations.

## Data availability statement

All datasets generated and analyzed in this study are available in the Supplementary material, further inquiries can be directed to the corresponding author.

## Author contributions

DW designed and supervised the study, commented on the data and its interpretation, and revised the content critically. ZZ and CC did the literature search, set up the database, and did all statistical analyses. ZZ and AP co-drafted the first version of the article. CC and BK helped with checking data and did the figures. All authors contributed to review and revision and approved the final manuscript as submitted and agreed to be accountable for all aspects of the work.

## Funding

This study was supported by the Science and Technology Commission of Shanghai Municipality (number: 22010503800).

## Conflict of interest

The authors declare that the research was conducted in the absence of any commercial or financial relationships that could be construed as a potential conflict of interest.

## Publisher's note

All claims expressed in this article are solely those of the authors and do not necessarily represent those of their affiliated organizations, or those of the publisher, the editors and the reviewers. Any product that may be evaluated in this article, or claim that may be made by its manufacturer, is not guaranteed or endorsed by the publisher.
