# Supplementary material for: A comparison of perceptual anticipation in combat sports between experts and non-experts: A systematic review and meta-analysis
Source: Front Psychol. 2022 Oct 28;13:961960. doi: 10.3389/fpsyg.2022.961960 (PMC9650920; doi:10.3389/fpsyg.2022.961960)
Supplement: Supplementary file 1 [file Data_Sheet_1.DOCX]

**Supplementary information**

# Table S1. Search strategy for the seven databases

| **Database** | **Step** | **Search strategy** | **Number of articles** |
| --- | --- | --- | --- |
| PubMed | #1 | anticipation[ti] OR prediction[ti] OR decision-making[ti] OR expert[ti] OR nonexpert[ti] OR expertise[ti] OR "visual search"[ti] OR "information processing"[ti] OR novice[ti] OR "cognitive characteristics"[ti] OR expertise[ti] OR cue use[ti] OR "eye movement"[ti] OR eye-tracking[ti] OR occlusion[ti] OR ((perceptual[ti] OR cognitive[ti]) AND skill*[ti]) OR ((visual[ti] OR spatial[ti]) AND attention[ti]) OR (reaction[ti] AND time*[ti]) | 211,033 |
|  | #2 | combating[ti] OR “combat sport”[ti] OR boxing[ti] OR boxer[ti] OR fencing[ti] OR fencer[ti] OR judo[ti] OR jiu-jitsu[ti] OR judoka[ti] OR wrestling[ti] OR wrestler[ti] OR karate[ti] OR karateka[ti] OR TaeKwonDo[ti] OR kickboxing[ti] OR “martial arts”[ti] OR Muay Thai[ti] OR kung-fu[ti] OR pankration[ti] | 5,416 |
|  | #3 | -2021/12/31 | - |
|  | #4 | Language: English | - |
|  | #5 | #1 **AND** #2 **AND** #3 **AND** #4 | 53 |
| Web of Science | #1 | TI = (anticipation OR prediction OR decision-making OR expert OR nonexpert OR expertise OR "visual search" OR "information processing" OR "cognitive characteristics" OR "cue use" OR "eye movement" OR eye-tracking OR occlusion OR ((perceptual OR cognitive) AND skill*) OR ((visual OR spatial) AND attention) OR (reaction AND time*)) | 876,383 |
|  | #2 | TI = (combating OR "combat sport" OR boxing OR boxer OR fencing OR fencer OR judo OR jiu-jitsu OR judoka OR wrestling OR wrestler OR karate OR karateka OR TaeKwonDo OR kickboxing OR "martial arts" OR Muay Thai OR kung-fu OR pankration) | 2,054,261 |
|  | #3 | -2021/12/31 | - |
|  | #4 | Language: English | - |
|  | #5 | Type: Article | - |
|  | #5 | #1 **AND** #2 **AND** #3 **AND** #4 **AND** #5 | 521 |
| EBSCO host | #1 | TI (anticipation OR prediction OR decision-making OR expert OR nonexpert OR expertise OR "visual search" OR "information processing" OR "cognitive characteristics" OR "cue use" OR "eye movement" OR eye-tracking OR occlusion OR ((perceptual OR cognitive) AND skill*) OR ((visual OR spatial) AND attention) OR (reaction AND time*)) | 811,506 |
|  | #2 | TI (combating OR "combat sport" OR boxing OR boxer OR fencing OR fencer OR judo OR jiu-jitsu OR judoka OR wrestling OR wrestler OR karate OR karateka OR TaeKwonDo OR kickboxing OR martial arts OR Muay Thai OR kung-fu OR pankration) | 100,226 |
|  | #3 | -2021/12/31 | - |
|  | #4 | Language: English | - |
|  | #5 | #1 **AND** #2 **AND** #3 **AND** #4 | 374 |
| EBSCO-SPORTDiscus | #1 | TI (anticipation OR prediction OR decision-making OR expert OR nonexpert OR expertise OR "visual search" OR "information processing" OR "cognitive characteristics" OR "cue use" OR "eye movement" OR eye-tracking OR occlusion) | 12,276 |
|  | #2 | TI (combating OR "combat sport" OR boxing OR boxer OR fencing OR fencer OR judo OR jiu-jitsu OR judoka OR wrestling OR wrestler OR karate OR karateka OR TaeKwonDo OR kickboxing OR martial arts OR Muay Thai OR kung-fu OR pankration) | 16,687 |
|  | #3 | -2021/12/31 | - |
|  | #4 | Language: English | - |
|  | #5 | #1 **AND** #2 **AND** #3 **AND** #4 | 81 |
| CNKI | #1 | anticipation + prediction + decision-making + expert + nonexpert + expertise + "visual search" + "information processing" + "cognitive characteristics" + "cue use" + "eye movement" + eye-tracking + occlusion | - |
|  | #2 | combating + "combat sport" + boxing + boxer + fencing + fencer + judo + jiu-jitsu + judoka + wrestling + wrestler + karate + karateka + TaeKwonDo + kickboxing + martial arts + Muay Thai + kung-fu + pankration | - |
|  | #3 | -2021/12/31 | - |
|  | #4 | #1 **AND** #2 **AND** #3 | 229 |
| Wanfang | #1 | anticipation OR prediction OR decision-making OR expert OR nonexpert OR expertise OR "visual search" OR "information processing" OR "cognitive characteristics" OR "cue use" OR "eye movement" OR eye-tracking OR occlusion | - |
|  | #2 | combating OR "combat sport" OR boxing OR boxer OR fencing OR fencer OR judo OR jiu-jitsu OR judoka OR wrestling OR wrestler OR karate OR karateka OR TaeKwonDo OR kickboxing OR martial arts OR Muay Thai OR kung-fu OR pankration | - |
|  | #3 | -2021/12/31 | - |
|  | #4 | #1 **AND** #2 **AND** #3 | 194 |
| CQVIP | #1 | anticipation OR prediction OR decision-making OR expert OR nonexpert OR expertise OR "visual search" OR "information processing" OR "cognitive characteristics" OR "cue use" OR "eye movement" OR eye-tracking OR occlusion | - |
|  | #2 | combating OR "combat sport" OR boxing OR boxer OR fencing OR fencer OR judo OR jiu-jitsu OR judoka OR wrestling OR wrestler OR karate OR karateka OR TaeKwonDo OR kickboxing OR martial arts OR Muay Thai OR kung-fu OR pankration | - |
|  | #3 | -2021/12/31 | - |
|  | #4 | #1 **AND** #2 **AND** #3 | 83 |

# Table S2. Inclusion and exclusion criteria for the systematic review.

| **Inclusion criteria** |
| --- |
| 1. Studies on perceptual anticipation in combat sports with the expert/non-expert paradigm. |
| 2. Quantitative measurements of the variables related to perceptual anticipation (e.g., reaction times, response accuracy, number and duration of fixations) using an experimental apparatus. |
| 3. Studies that reported the mean and standard error of the variables. |
| 4. Full texts of the studies were available. |
| **Exclusion criteria** |
| 1. The mean and standard error of the variables were not reported. |
| 2. Full texts of the studies were available. |
| 3. Abstracts from congress meetings or conference proceedings, study protocols, news outlets, commentaries, dissertations, reviews, and case reports were excluded. |

# Table S3. Modified MINORS system for evaluating the risk of bias of the included studies

| Item | Score |
| --- | --- |
| 1. The study’s aim is stated | 0=unreported, 1=reported but insufficient, 2=reported and sufficient |
| 2. Participants at different levels included | 0=unreported, 1=reported but insufficient, 2=reported and sufficient |
| 3. Appropriate method for collecting data | 0=unreported, 1=reported but insufficient, 2=reported and sufficient |
| 4. Endpoint appropriate for the study’s aim | 0=unreported, 1=reported but insufficient, 2=reported and sufficient |
| 5. Unbiased evaluation of endpoints | 0=unreported, 1=reported but insufficient, 2=reported and sufficient |
| 6. A comparable control group | 0=unreported, 1=reported but insufficient, 2=reported and sufficient |
| 7. Baseline equivalence of groups | 0=unreported, 1=reported but insufficient, 2=reported and sufficient |
| 8. Statistical analyses adapted to the study’s design | 0=unreported, 1=reported but insufficient, 2=reported and sufficient |

# Table S4. Scores of the included studies according to MINORS

| Item | Ottoboni et al. | Bianco et al. | Ripoll et al. | Allerdissen et al. | Chan et al. | Rosalie et al. | Piras et al. | Mori et al. | Wang et al. | Zhao et al. | Liu et al. | Feng et al. | Feng et al. | Liu et al. | He et al. | Liu et al. | Zhao et al. | Feng et al. | Milazzo et al. | Walliams et al. | Li et al. | Wang et al. | Fu et al. |
| --- | --- | --- | --- | --- | --- | --- | --- | --- | --- | --- | --- | --- | --- | --- | --- | --- | --- | --- | --- | --- | --- | --- | --- |
| 1. The study’s aim is stated | 2 | 2 | 2 | 2 | 2 | 2 | 2 | 2 | 2 | 2 | 2 | 2 | 2 | 2 | 2 | 2 | 2 | 2 | 2 | 2 | 2 | 2 | 2 |
| 2. Participants at different levels included | 2 | 2 | 2 | 2 | 2 | 2 | 2 | 2 | 2 | 2 | 2 | 2 | 2 | 2 | 2 | 2 | 2 | 2 | 2 | 2 | 2 | 2 | 2 |
| 3. Appropriate method for collecting data | 2 | 2 | 2 | 2 | 2 | 2 | 2 | 2 | 2 | 2 | 2 | 2 | 2 | 2 | 2 | 2 | 2 | 2 | 2 | 2 | 2 | 2 | 2 |
| 4. Endpoint appropriate for the study’s aim | 2 | 2 | 2 | 2 | 2 | 2 | 2 | 2 | 2 | 2 | 2 | 2 | 2 | 2 | 2 | 2 | 2 | 2 | 2 | 2 | 2 | 2 | 2 |
| 5. Unbiased evaluation of endpoints | 1 | 2 | 2 | 2 | 2 | 2 | 2 | 2 | 1 | 2 | 2 | 2 | 1 | 2 | 2 | 1 | 1 | 2 | 2 | 1 | 1 | 2 | 2 |
| 6. A comparable control group | 2 | 2 | 2 | 2 | 2 | 2 | 2 | 2 | 2 | 2 | 2 | 2 | 2 | 2 | 2 | 2 | 2 | 2 | 2 | 2 | 2 | 2 | 2 |
| 7. Baseline equivalence of groups | 2 | 2 | 1 | 2 | 2 | 1 | 2 | 2 | 2 | 2 | 2 | 2 | 2 | 2 | 2 | 2 | 2 | 1 | 1 | 2 | 2 | 2 | 2 |
| 8. Statistical analyses adapted to the study’s design | 2 | 1 | 2 | 1 | 2 | 1 | 1 | 1 | 1 | 1 | 1 | 1 | 1 | 1 | 1 | 1 | 1 | 1 | 1 | 1 | 2 | 1 | 1 |
| Total | 15 | 15 | 16 | 15 | 16 | 14 | 15 | 15 | 14 | 15 | 15 | 15 | 14 | 15 | 15 | 14 | 14 | 14 | 14 | 14 | 15 | 15 | 15 |

| Item | Del Percio et al. | Shih et al. | Cojocariu et al. | Muinos et al. | Babadi et al. | Gutierrez-Davila et al. | Martinez de Que et al. | Chung et al. | Bianco et al. | Del Percio et al. | Del Percio et al. | Chen et al. | Williams et al. | Johne et al. | Fontani et al. | Mouelhi Guizani et al. | Williams et al. | Lesiakowski et al. | Zbigniew et al. |
| --- | --- | --- | --- | --- | --- | --- | --- | --- | --- | --- | --- | --- | --- | --- | --- | --- | --- | --- | --- |
| 1. The study’s aim is stated | 2 | 2 | 2 | 2 | 2 | 2 | 2 | 2 | 2 | 2 | 2 | 2 | 2 | 2 | 2 | 2 | 2 | 2 | 2 |
| 2. Participants at different levels included | 2 | 2 | 2 | 2 | 2 | 2 | 2 | 2 | 2 | 2 | 2 | 2 | 2 | 2 | 2 | 2 | 2 | 2 | 2 |
| 3. Appropriate method for collecting data | 2 | 2 | 2 | 2 | 2 | 2 | 2 | 2 | 2 | 2 | 2 | 2 | 2 | 2 | 2 | 2 | 2 | 2 | 2 |
| 4. Endpoint appropriate for the study’s aim | 2 | 2 | 2 | 2 | 2 | 2 | 2 | 2 | 2 | 2 | 2 | 2 | 2 | 2 | 2 | 2 | 2 | 2 | 2 |
| 5. Unbiased evaluation of endpoints | 1 | 2 | 2 | 2 | 1 | 2 | 2 | 1 | 1 | 2 | 2 | 2 | 2 | 2 | 2 | 1 | 2 | 2 | 2 |
| 6. A comparable control group | 2 | 2 | 2 | 2 | 2 | 2 | 2 | 2 | 2 | 2 | 2 | 2 | 2 | 2 | 2 | 2 | 2 | 2 | 2 |
| 7. Baseline equivalence of groups | 2 | 2 | 1 | 2 | 2 | 2 | 1 | 2 | 2 | 2 | 2 | 2 | 1 | 2 | 2 | 2 | 2 | 2 | 1 |
| 8. Statistical analyses adapted to the study’s design | 1 | 1 | 1 | 1 | 1 | 1 | 1 | 1 | 2 | 1 | 1 | 2 | 1 | 1 | 1 | 1 | 1 | 2 | 1 |
| Total | 14 | 15 | 14 | 15 | 14 | 15 | 14 | 14 | 15 | 15 | 15 | 16 | 14 | 15 | 15 | 14 | 15 | 16 | 14 |

# Table S5. Summary of the included studies

| **Reference** | **Sport type** | **Participants** | **Number of participants** | **Stimulus material(s)** | **Experimental task** | **Indicator(s)** | **Value (mean±SD)** |
| --- | --- | --- | --- | --- | --- | --- | --- |
| Ottoboni et al. (2014)^1^ (English) | Boxing | Boxer | 36 (Exp: 12, Mid: 8, Non-exp: 15) | Static | Participants were seated in front of the computer screen and directed to code the color of the gloves by pressing the “x” key on the keyboard with their left hand and the “.” key with their right hand. | Accuracy (%) | Right punched-right:  - Exp: 96.41±1.24; Mid: 96.25±1.16; Non-exp: 93.11±1.27  Left punched-right:  - Exp: 94.62±1.36; Mid: 98.33±1.11; Non-exp: 95.33±1.21  Right punched-left:  - Exp: 95.13±1.26; Mid: 97.50±1.67; Non-exp: 96.22±0.93  Left punched-left  - Exp: 96.67±1.26; Mid: 96.25±1.71; Non-exp: 95.56±0.98 |
|  |  |  | 36 (Exp: 12, Mid: 8, Non-exp: 15) |  |  | Reaction time (ms) | Exp: 448; Mid: 372; Non-exp: 410 |
| Bianco et al. (2017)^2^  (English) | Boxing | Boxer | 39 (Exp: 13, Non-exp: 13) | Dynamic | They were asked to be accurate in discriminating the stimuli and to respond as soon as possible when the target (go stimuli) appeared on the screen, and to withhold the response when the non-target appeared. | Reaction time (ms) | Exp: 418±55; Non-exp: 480±48 |
|  | Fencing | Fencer | 39 (Exp: 13, Non-exp: 13) | Dynamic |  | Reaction time (ms) | Exp: 402±55; Non-exp: 480±48 |
| Ripoll et al. (1995)^3^  (English) | Boxing | Boxer | 18 (Exp: 6, Mid: 6, Non-exp: 6) | Dynamic | Subjects had to respond to different boxing areas on the screen by manipulating a joystick. | Accuracy (%) | Exp: 93.3; Mid: 86.6; Non-exp: 88.3 |
|  |  |  | 18 (Exp: 6, Mid: 6, Non-exp: 6) |  |  | Reaction time (ms) | Exp: 546±103.2; Mid: 574±88.2; Non-exp: 534±56.8 |
| Allerdissen et al. (2017)^4^ (English) | Fencing | Fencer | 32 (Exp: 15, Non-exp: 17) | Dynamic | Participants were asked to press the assigned response keys when the stimuli would stop at an unknown point. | Accuracy (%) | First point of occlusion/audio-visual:  - Exp: 49.6±6.3; Non-exp: 48.9±4.3  First point of occlusion/visual:  - Exp: 48.8±5.6; Non-exp: 51.2±6.9  First point of occlusion/audio:  - Exp: 50.4±7.7; Non-exp: 50.6±5.3  Second point of occlusion/audio-visual:  - Exp: 79.7±11.8; Non-exp: 55.6±11.5  Second point of occlusion/visual:  - Exp: 80.6±12.5; Non-exp: 64.6±11.6  Second point of occlusion/audio:  - Exp: 51.7±5.2; Non-exp: 51.0±5.0  Third point of occlusion/audio-visual:  - Exp: 98.6±3.2; Non-exp: 71.7±24.2  Second point of occlusion/visual:  - Exp: 97.1±3.8; Non-exp: 97.3±4.3  Second point of occlusion/audio:  - Exp: 61.7±12.2; Non-exp: 50.6±9.0  Fourth point of occlusion/audio-visual:  - Exp: 99.7±0.7; Non-exp: 96.2±4.8  Second point of occlusion/visual:  - Exp: 99.4±1.0; Non-exp: 98.9±2.7  Second point of occlusion/audio:  - Exp: 94.3±3.9; Non-exp: 93.4±6.6 |
| Chan et al. (2011)^5^ (English) | Fencing | Fencer | 60 (Exp: 15, Non-exp: 15) | Dynamic | Participants performed an SRT task by responding to all stimuli, responding only to the “go” signals while not responding to the “no-go” signals. | Reaction time (ms) | Simple RT/average-fit fencer:  - Exp: 293.08±29.23; Non-exp: 311.63±65.92  Simple RT/high-fit fencer:  - Exp: 300.37±37.55; Non-exp: 313.99±49.41  Go or no-Go RT/average-fit fencer:  - Exp: 300.37±37.55; Non-exp: 313.99±49.41  Go or no-Go RT/high-fit fencer:  - Exp: 448.85±63.50; Non-exp: 430.22±67.6 |
| Rosalie et al. (2013)^6^ (English) | Karate | Karate athlete | 21 (Exp: 8, Mid: 6, Non-exp: 7) | In situ | Participants stood facing an opponent and then attempted to block attacks (kicks and punches). | Accuracy (%) | First point of occlusion:  - Exp: 37.62±2.27; Mid: 33.11±3.84; Non-exp: 23.25±2.00  Second point of occlusion:  - Exp: 41.29±2.17; Mid: 27.43±1.17; Non-exp: 21.24±2.84  Third point of occlusion:  - Exp: 60.00±2.27; Mid: 33.1±3.84; Non-exp: 23.3±2.0  Fourth point of occlusion:  - Exp: 85.05±3.00; Mid: 68.85±3.67; Non-exp: 48.98±5.01 |
| Piras et al. (2014)^7^ (English) | Judo | Judoka | 20 (Exp: 9, Non-exp: 11) | In situ | Subjects used a target-directed strategy or tried to perceive whole body movements by fixating on non-critical body parts. | No. of fixations | Lapel attack:  - Exp: 3.65±0.13; Non-exp: 3.98±0.14  Lapel defense:  - Exp: 3.68±0.13; Non-exp: 4.13±0.13  Sleeve attack:  - Exp: 3.75±0.13; Non-exp: 4.51±0.17  Sleeve defense:  - Exp: 3.59±0.13; Non-exp: 3.79±0.09  Total:  - Exp: 3.67±0.07; Non-exp: 4.04±0.06 |
| Mori et al. (2002)^8^ (English) | Karate | Karate athlete | 13 (Exp: 6, Non-exp: 7) | Dynamic | Choice RT task: Participants had to indicate the offensive actions of the body level or the dot position. Simple RT task: They had to respond when the offensive action started from the ready stance or a dot appeared on the display. | Reaction time (ms) | Video Choice RT  - Exp: 551.3±10.7; Non-exp: 656.8±7.7  Dot Choice RT  - Exp: 270.4±6.1; Non-exp: 297.8±7.7  Video Simple RT  - Exp: 225.5±3.1; Non-exp: 245.2±10.7  Dot Simple RT  - Exp: 234.2±3.1; Non-exp: 244.7±9.19 |
|  |  |  |  |  |  | Accuracy (%) | Exp: 96.0±2.0; Non-exp: 80.0±7.8 |
| Wang et al. (2011)^9^ (Chinese) | Taekwondo | Taekwondo athletes | 94 (Exp: 30, Mid: 32, Non-exp: 32) | Static | Participants were asked to make intuitive decisions about text information. | Accuracy (%) | 20% prior probability/male  - Exp: 78.00±3.26; Mid: 66.15±8.44; Non-exp: 56.94±8.07  20% prior probability/female  - Exp: 71.11±9.79; Mid: 63.68±12.34; Non-exp: 52.14±12.67  50% prior probability/male  - Exp: 77.5±5.44; Mid: 71.54±9.44; Non-exp: 57.78±8.95  50% prior probability/female  - Exp: 73.61±8.19; Mid: 67.11±8.39; Non-exp: 57.5±12.67  80% prior probability/male  - Exp: 86.25±4.33; Mid: 78.85±5.46; Non-exp: 64.72±9.31  80% prior probability/female  - Exp: 80.83±7.72; Mid: 75.26±7.9; Non-exp: 65.3±10.46 |
|  | Taekwondo | Taekwondo athletes | 94 (Exp: 30, Mid: 32, Non-exp: 32) | Dynamic | Participants were asked to make intuitive decisions about text information. | Accuracy (%) | 20% prior probability/male  - Exp: 77.94±5.02; Mid: 69.72±6.96; Non-exp: 60.95±9.43  20% prior probability/female  - Exp: 76.58±5.28; Mid: 71.94±13.19; Non-exp: 57.86±10.87  50% prior probability/male  - Exp: 83.24±3.93; Mid: 73.89±8.14; Non-exp: 60.71±7.79  50% prior probability/female  - Exp: 79.21±5.59; Mid: 74.72±4.69; Non-exp: 60.71±6.16  80% prior probability/male  - Exp: 90.00±3.95; Mid: 78.06±5.98; Non-exp: 67.38±9.95  80% prior probability/female  - Exp: 86.05±7.18; Mid: 76.94±12.26; Non-exp: 67.14±13.97 |
|  | Taekwondo | Taekwondo athletes | 94 (Exp: 30, Mid: 32, Non-exp: 32) | Static | Participants were asked to make intuitive decisions about text information. | Reaction time (ms) | 20% prior probability/male  - Exp: 520.08±160.85; Mid: 633.23±153.36; Non-exp: 693.56±204.10  20% prior probability/female  - Exp: 575.94±125.6; Mid: 574.21±228.50; Non-exp: 630.71±308.48  50% prior probability/male  - Exp: 456.67±168.51; Mid: 546.00±193.95; Non-exp: 625.56±223.12  50% prior probability/female  - Exp: 480.22±105.52; Mid: 534.00±154.63; Non-exp: 516.64±195.37  80% prior probability/male  - Exp: 355.75±112.89; Mid: 392.69±128.89; Non-exp: 582.33±133.29  80% prior probability/female  - Exp: 364.61±119.85; Mid: 412.00±262.58; Non-exp: 420.36±163.62 |
|  | Taekwondo | Taekwondo athletes | 94 (Exp: 30, Mid: 32, Non-exp: 32) | Dynamic | Participants were asked to make intuitive decisions about text information. | Reaction time (ms) | 20% prior probability/male  - Exp: 455.76±89.83; Mid: 528.72±109.33; Non-exp: 669.12±141.42  20% prior probability/female  - Exp: 511.26±134.12; Mid: 576.39±271.50; Non-exp: 622.36±168.68  50% prior probability/male  - Exp: 364.65±112.41; Mid: 465.67±136.33; Non-exp: 552.19±190.14  50% prior probability/female  - Exp: 364.21±67.77; Mid: 446.39±195.40; Non-exp: 560.07±188.55  80% prior probability/male  - Exp: 280.94±109.67; Mid: 402.56±114.37; Non-exp:444.38±177.83  80% prior probability/female  - Exp: 312.84±93.83; Mid:356.28±142.89; Non-exp: 439.43±163.69 |
| Zhao et al. (2014)^10^ (Chinese) | Sanda | Sanda athletes | 28 (Exp: 16, Non-exp: 12) | Dynamic | Participants were asked to predict the attack and respond by pressing a button when watching the video (picture). | Reaction time (ms) | Exp: 1479.35±129.07; Non-exp: 1622.15±125.66 |
|  |  |  | 28 (Exp: 16, Non-exp: 12) | Static |  | Reaction time (ms) | Exp: 992.19±129.07; Non-exp: 1160.75±265.09 |
|  |  |  | 28 (Exp: 16, Non-exp: 12) | Dynamic |  | Accuracy (%) | Exp: 63.18±8.25; Non-exp: 53.19±6.9 |
|  |  |  | 28 (Exp: 16, Non-exp: 12) | Static |  | Accuracy (%) | Exp: 1.67±3.8; Non-exp: 50.66±6.7 |
| Feng et al. (2015)^11^ (Chinese) | Fencing | Fencer | 33 (Exp: 8, Mid: 14, Non-exp: 11) | Dynamic | Subjects were asked to follow the screen prompt to complete the subsequent button response. | Reaction time (ms) | Exp: 643.81±179.44; Mid:535.96±109.93; Non-exp: 554.16±176.58 |
|  |  |  | 33 (Exp: 8, Mid: 14, Non-exp: 11) |  |  | Accuracy (%) | Exp: 65.03±8.90; Mid:60.92±10.39; Non-exp: 63.36±10.06 |
| Feng et al. (2015)^12^ (Chinese) | Fencing | Fencer | 33 (Exp: 8, Mid: 14, Non-exp: 11) | Static | Subjects were asked to judge the tactics the opponent should adopt according to the tactical intention of the attacking team members in the video. | Reaction time (ms) | Exp: 620.82±107.77; Mid: 642.80±50.68; Non-exp: 628.58±65.71 |
|  |  |  | 33 (Exp: 8, Mid: 14, Non-exp: 11) |  |  | Accuracy (%) | Exp: 76.33±5.79; Mid: 75.46±4.19; Non-exp: 74.4±7.3 |
| Liu et al. (2010)^13^ (Chinese) | Sanda | Sanda athletes | 20 (Exp: 10 Non-exp: 10) | Dynamic | Participants used visual search to find key clues to video movements. | Mean duration of fixation (s) | Exp: 3.32±1.56; Non-exp: 1.68±1.62 |
|  |  |  | 20 (Exp: 10 Non-exp: 10) |  |  | Frequency of fixation (times/s) | Exp: 82.95±39.05; Non-exp: 41.96±40.53 |
| He et al. (2016)^14^ (Chinese) | Sanda | Sanda athletes | 24 (Exp: 12, Non-exp: 12) | Static | Participants were asked to judge whether the attackers in the pictures used defensive tactics or continued to attack. | Accuracy (%) | Exp: 80.2; Non-exp: 58.3 |
|  | Sanda | Sanda athletes | 24 (Exp: 12, Non-exp: 12) | Static | Participants were asked to judge whether the attackers in the pictures used defensive tactics or continued to attack. | No. of fixations (times) | Head:  - Exp: 5.42±1.62; Non-exp: 3.85±1.31  Chest:  - Exp: 9.75±2.30; Non-exp: 4.92±1.42  Upper limb:  - Exp: 4.25±2.09; Non-exp: 8.46±1.83  Leg:  - Exp: 7.33±2.71; Non-exp: 8.85±3.02  Other:  - Exp: 2.08±0.79; Non-exp: 6.38±1.76 |
|  | Sanda | Sanda athletes | 24 (Exp: 12, Non-exp: 12) | Static | Participants were asked to judge whether the attackers in the pictures used defensive tactics or continued to attack. | Mean duration of fixation(s) | Head:  - Exp: 2.01±0.71; Non-exp: 0.9±0.34  Chest:  - Exp: 2.19±0.71; Non-exp: 1.53±0.52  Upper limb:  - Exp: 1.30±0.55; Non-exp: 3.60±1.02  Leg:  - Exp: 2.46±0.76; Non-exp: 2.55±0.98  Other:  - Exp: 0.44±0.30; Non-exp: 1.92±0.60 |
| Liu et al. (2017)^15^ (Chinese) | Taekwondo | Taekwondo athletes | 40 (Exp: 20, Non-exp: 20) | Static | Participants were required to find the same letter in the matrix under the premise of a correct judgment by pressing a key to respond. | Reaction time (ms) | Exp: 1378.08±30.81; Non-exp: 1539.46±30.07 |
| Zhao et al. (2010)^16^ (Chinese) | Sanda | Sanda athletes | 26 (Exp: 13, Non-exp: 13) | Static | Participants were asked to respond to feature search images in a timely manner. | Reaction time (ms) | Experiment 1:  - Exp: 717.91±75.65; Non-exp: 886.14±58.30  Experiment 2:  - Exp: 729.88±71.95; Non-exp: 860.16±78.12  Experiment 3:  - Exp: 728.52±94.78; Non-exp: 841.78±103.06 |
| Feng et al. (2018)^17^ (Chinese) | Boxing | Boxer | 30 (Exp: 15, Non-exp: 15) | Static | Participants were asked to respond to the key according to the position represented by the red circle in the picture. | Reaction time (ms) | Head:  - Exp: 433.56±35.7; Non-exp: 485.16±52.03  Chest:  - Exp: 438.86±41.82; Non-exp: 485.84±51.9  Left rib:  - Exp: 415.11±37.34; Non-exp: 471.07±74.87  Right rib:  - Exp: 380.07±36.50; Non-exp: 442.10±95.45 |
|  |  |  | 30 (Exp: 15, Non-exp: 15) | Static |  | Accuracy (%) | Head:  - Exp: 96.50±3.87; Non-exp: 90.33±13.75  Chest:  - Exp:89.17±4.88; Non-exp: 86.50±9.94  Left rib:  - Exp: 89.49±5.98; Non-exp: 76.67±22.27  Right rib:  - Exp: 95.83±4.3; Non-exp: 80.67±29.89 |
| Milazzo et al. (2015)^18^ (English) | Karate | Karate athlete | 28 (Exp: 14, Non-exp: 14) | In situ | Participants were required to react and make decisions about various attacks in different fighting scenarios against a standardized expert opponent. | Reaction time (ms) | Exp: 268.1±42.3; Non-exp: 354.4±34.2 |
|  |  |  | 28 (Exp: 14, Non-exp: 14) | In situ |  | Accuracy (%) | Action1:  - Exp: 35.5; Non-exp: 25.0  Action2:  - Exp: 64.5; Non-exp: 18.0  Action3:  - Exp: 64.5; Non-exp: 28.5  Action4:  - Exp: 71.5; Non-exp: 35.5  Action5:  - Exp: 82.0; Non-exp: 32.0  Action6:  - Exp: 85.5; Non-exp: 32.0 |
|  |  |  | 28 (Exp: 14, Non-exp: 14) | Dynamic |  | Mean duration of fixation(s) | Exp: 1.03±0.43; Non-exp: 0.51±0.07 |
|  |  |  | 28 (Exp: 14, Non-exp: 14) | Dynamic |  | No. of fixations (times) | Exp: 4.10±0.50; Non-exp: 8.60±0.50 |
| Walliams et al. (2000)^19^ (English) | Fencing | Fencer | 6 (Exp: 3, Non-exp: 3) | Dynamic | Participants were asked to strike the primary target when the stimulus light flashed on. | Reaction time (ms) | Exp: 333±128; Non-exp: 613±62 |
| Li et al. (2010)^20^ (Chinese) | Fencing | Fencer | 24 (Exp: 12, Non-exp: 12) | Dynamic | Participants were asked to judge the landing point by watching the video of the fencing attack and pressing the appropriate key. | Accuracy (%) | Exp: 51.93; Non-exp: 26.22 |
|  |  |  | 24 (Exp: 12, Non-exp: 12) | Dynamic |  | Reaction time (ms) | Exp: 1570.00±167.84; Non-exp: 1821.1±290.34 |
|  |  |  | 24 (Exp: 12, Non-exp: 12) | Dynamic |  | No. of fixations (times) | Exp: 5.74±0.97; Non-exp: 7.73±1.46 |
|  |  |  | 24 (Exp: 12, Non-exp: 12) | Dynamic |  | Frequency of fixation (times/s) | Exp: 1.33±0.49; Non-exp: 2.25±0.97 |
| Wang et al. (2009)^21^ (Chinese) | Taekwondo | Taekwondo athletes | 75 (Exp: 27, Mid: 22, Non-exp: 26) | Dynamic | Participants were required to press the key according to the instructions. | Reaction time (ms) | 20% prior probability/male:  - Exp: 565.59±244.37; Mid: 433.23±140.92; Non-exp: 539.08±276.00  50% prior probability/male:  - Exp: 586.30±169.13; Mid: 469.23±146.75; Non-exp: 571.04±268.23  80% prior probability/male:  - Exp: 391.78±182.89; Mid: 415.09±189.03; Non-exp: 491.81±263.91 |
|  | Taekwondo | Taekwondo athletes | 70 (Exp: 21, Mid: 28, Non-exp: 21) | Dynamic | Participants were required to press the key according to the instructions. | Reaction time (ms) | 20% prior probability/female:  - Exp: 457.05±162.85; Mid: 502.04±150.33; Non-exp: 533.33±162.86  50% prior probability/female:  - Exp: 497.86±144.18; Mid: 477.25±151.15; Non-exp: 481.76±171.97  80% prior probability/female:  - Exp: 369.00±140.30; Mid: 416.61±157.39; Non-exp: 472.57±184.39 |
|  | Taekwondo | Taekwondo athletes | 75 (Exp: 27, Mid: 22, Non-exp: 26) | Dynamic | Participants were required to press the key according to the instructions. | Accuracy (%) | 20% prior probability/male:  - Exp: 73.33±6.58; Mid: 64.66±6.47; Non-exp: 54.13±6.85  50% prior probability/male:  - Exp: 75.22±5.92; Mid: 65.86±4.86; Non-exp: 54.63±6.59  80% prior probability/male:  - Exp: 82.76±3.97; Mid: 73.07±5.92; Non-exp: 59.33±9.07 |
|  | Taekwondo | Taekwondo athletes | 70 (Exp: 21, Mid: 28, Non-exp: 21) | Dynamic | Participants were required to press the key according to the instructions. | Accuracy (%) | 20% prior probability/female:  - Exp: 75.00±6.22; Mid: 62.79±9.07; Non-exp: 52.86±11.08  50% prior probability/female:  - Exp: 75.48±5.95; Mid: 66.25±7.92; Non-exp: 53.69±7.81  80% prior probability/female:  - Exp: 83.38±4.29; Mid: 70.63±6.11; Non-exp: 56.79±9.73 |
| Fu et al. (2010)^22^ (Chinese) | Fencing | Fencer | 36 (Exp: 9, Mid: 19, Non-exp: 8) | Static | Participants were asked to respond to different types of tasks on the computer. | Reaction time (ms) | Fleuret/simple reaction  - Exp: 216.56±15.31; Mid: 222.16±8.55; Non-exp: 235.13±10.49  Fleuret/discrimination reaction  - Exp: 311.33±23.47; Mid: 347.21±31.10; Non-exp: 380.00±50.43  Fleuret/Complex stimulus type 2  - Exp: 357.78±33.76; Mid: 366.16±27.17; Non-exp: 386.00±30.76  Fleuret/Complex stimulus type 4  - Exp: 498.44±43.15; Mid: 546.11±43.08; Non-exp: 582.50±61.30  Fleuret/reverse reaction  - Exp: 577.78±73.9; Mid: 638.68±82.05; Non-exp: 714.75±101.43 |
|  | Fencing | Fencer | 36 (Exp: 5, Mid: 17, Non-exp: 14) | Static | Participants were asked to respond to different types of tasks on the computer. | Reaction time (ms) | Saber fencing/simple reaction  - Exp: 224.60±21.93; Mid: 228.41±16.10; Non-exp: 234.29±11.07  Fleuret/discrimination reaction  - Exp: 318.80±37.24; Mid: 338.18±48.07; Non-exp: 385.64±29.77  Saber fencing/Complex stimulus type 2  - Exp: 356.00±47.79; Mid: 363.00±25.62; Non-exp: 392.14±37.91  Saber fencing/Complex stimulus type 4  - Exp: 553.40±130.86; Mid: 551.82±44.32; Non-exp: 599.70±66.50  Saber fencing/reverse reaction  - Exp: 599.40±129.29; Mid: 623.65±73.17; Non-exp: 699.43±90.07 |
|  | Fencing | Fencer | 36 (Exp: 8, Mid: 15, Non-exp: 20) | Static | Participants were asked to respond to different types of tasks on the computer. | Reaction time (ms) | Epee/simple reaction  - Exp: 234.75±25.76; Mid: 226.53±20.05; Non-exp: 227.25±17.31  Epee/discrimination reaction  - Exp: 331.62±44.67; Mid: 327.13±42.76; Non-exp: 348.50±38.70  Epee/Complex stimulus type 2  - Exp: 373.75±50.82; Mid: 366.8±41.20; Non-exp: 363.30±20.94  Epee/Complex stimulus type 4  - Exp: 550.25±101.01; Mid: 570.27±64.58; Non-exp: 586.10±53.74  Epee/reverse reaction  - Exp: 605.75±103.06; Mid: 661.00±108.68; Non-exp: 639.3±125.88 |
| Del Percio et al. (2009)^23^ (English) | Karate | Karate athletes | 22 (Exp: 11, Non-exp: 11) | Static | Participants were requested to immediately respond by pressing buttons on the keyboard with the left or right finger, respectively. | Accuracy (%) | Right movement  - Exp: 96.1±1.5; Non-exp: 95.8±1  Left movement  - Exp: 95±1.3; Non-exp: 94.2±1.2 |
|  | Fencing | Fencer | 22 (Exp: 11, Non-exp: 11) | Static |  | Accuracy (%) | Right movement  - Exp: 91.6±1.5; Non-exp: 95.8±1  Left movement  - Exp: 91.8±1.3; Non-exp: 94.2±1.2 |
|  | Karate | Karate athletes | 22 (Exp: 11, Non-exp: 11) | Static | Participants were requested to immediately respond by pressing buttons on the keyboard with the left or right finger, respectively. | Reaction time (ms) | Right movement  - Exp: 394±19; Non-exp: 499±36  Left movement  - Exp: 399±26; Non-exp: 524±37 |
|  | Fencing | Fencer | 22 (Exp: 11, Non-exp: 11) | Static |  | Reaction time (ms) | Right movement  - Exp: 412±24; Non-exp: 499±36  Left movement  - Exp: 422±31; Non-exp: 524±37 |
| Shih et al. (2016)^24^ (English) | Taekwondo | Taekwondo athletes | 52 (Exp: 14, Non-exp: 14) | Static | Participants were asked to decide on the kick for the taekwondo set, a snatch for the weightlifting set, or an emotion for the facial expression set. | Reaction time (ms) | 25%  - Exp: 938±136; Non-exp: 1104± 277  50%  - Exp: 795±115; Non-exp: 950±205  75%  - Exp: 737±75; Non-exp: 869±202  100%  - Exp: 690±60; Non-exp: 745±131 |
|  |  |  |  |  |  | Accuracy (%) | 25%  - Exp: 69.62±10.18; Non-exp: 61.36±9.11  50%  - Exp: 86.9±12.44; Non-exp: 81.15±12.29  75%  - Exp: 96.83±5.85; Non-exp: 90.28±7.37  100%  - Exp: 95.62±8.63; Non-exp: 98.02±2.97 |
| Cojocariu et al.^25^ (2014) (English) | Judo | Judoka | 28 (Exp: 8, Non-exp: 20) | Dynamic | Participants were asked to use the same set of fingers attached and the dominant or non-dominant hand, and to press the red button. | Reaction time (ms) | Simple reaction time  - Exp: 233.6±5; Non-exp: 238.7±6.8  Choice reaction time [ms] dominant hand  - Exp: 404.1±11; Non-exp: 421.3±9.5  Choice reaction time [ms] non-dominant hand  - Exp: 409±13.6; Non-exp: 425.9±10.2  Choice reaction time [ms] dominant hand  - Exp: 391±11.9; Non-exp: 407.8±8.1  Choice reaction time [ms] non-dominant hand  - Exp: 395.3±18.6; Non-exp: 418.5±16.2 |
| Muiños et al. (2014)^26^ (English) | Judo | Judoka | 90 (Exp: 30, Non-exp: 30) | Dynamic | Participants were instructed to fixate on and view the stimuli binocularly, and to press a keyboard button when the stimulus appeared. | Reaction time (ms) | E3.250-Experiment 1  - Exp: 239.7±23.6; Non-exp: 250.07±20.99  E3.700-Experiment 1  - Exp: 242.9±27.8; Non-exp: 236.6±26.6  E3.1500-Experiment 1  - Exp: 244.1±23.8; Non-exp: 245.8±14.3  E6.250-Experiment 1  - Exp: 237.7±14.8; Non-exp: 240.9±14.2  E6.700-Experiment 1  - Exp: 230.2±14.9; Non-exp: 230.8±16.5  E6.1500-Experiment 1  - Exp: 243.8±24.4; Non-exp: 249.3±16  E12.250-Experiment 1  - Exp: 252.5±14.7; Non-exp: 259.9±16.4  E12.700-Experiment 1  - Exp: 241.5±15.8; Non-exp: 248.6±22.4  E12.1500-Experiment 1  - Exp: 248.1±20.1; Non-exp: 258.8±19.2 |
|  | Karate | Karate athletes | 90 (Exp: 30, Non-exp: 30) | Dynamic | Participants were instructed to fixate on and view the stimuli binocularly, and to press a keyboard button when the stimulus appeared. | Reaction time (ms) | E3.250-Experiment 1  - Exp: 241.7±28.8; Non-exp: 250.07±20.99  E3.700-Experiment 1  - Exp: 233±25.8; Non-exp: 236.6±26.6  E3.1500-Experiment 1  - Exp: 239.6±25; Non-exp: 245.8±14.3  E6.250-Experiment 1  - Exp: 230.5±14.8; Non-exp: 240.9±14.2  E6.700-Experiment 1  - Exp: 230.6±15.7; Non-exp: 230.8±16.5  E6.1500-Experiment 1  - Exp: 241.1±20.5; Non-exp: 249.3±16  E12.250-Experiment 1  - Exp: 242.7±13.3; Non-exp: 259.9±16.4  E12.700-Experiment 1  - Exp: 235.9±16.9; Non-exp: 248.6±22.4  E12.1500-Experiment 1  - Exp: 238.1±12.8; Non-exp: 258.8±19.2 |
|  | Karate | Karate athletes | 45 (Exp: 15, Non-exp: 15) | Dynamic | Participants were instructed to fixate on and view the stimuli binocularly, and to press a keyboard button when the stimulus appeared. | Reaction time (ms) | E3.250-Experiment 2  - Exp: 243.8±24.5; Non-exp: 282.5±12.5  - Exp: 247.1±24.5; Non-exp: 282.5±12.5  E3.700-Experiment 2  - Exp: 247±26.7; Non-exp: 264.9±24.4  - Exp: 251.1±29.2; Non-exp: 264.9±24.4  E3.1500-Experiment 2  - Exp: 252.6±20.9; Non-exp: 281.1±13.9  - Exp: 247.9±23.4; Non-exp: 281.1±13.9  E6.250-Experiment 2  - Exp: 241.4±16.7; Non-exp: 275.5±14.8  - Exp: 249.3±17.3; Non-exp: 275.5±14.8  E6.700-Experiment 2  - Exp: 236.9±17.1; Non-exp: 264±17.9  - Exp: 235.9±14.1; Non-exp: 264±17.9  E6.1500-Experiment 2  - Exp: 251.5±31; Non-exp: 285.5±10.9  - Exp: 248.1±21.2; Non-exp: 285.5±10.9  E12.250-Experiment 2  - Exp: 255.6±15.9; Non-exp: 287.4±13.8  - Exp: 257.3±14.9; Non-exp: 287.4±13.8  E12.700-Experiment 2  - Exp: 249±18.9; Non-exp: 289.1±22.1  - Exp: 245.3±15.6; Non-exp: 289.1±22.1  E12.1500-Experiment 2  - Exp: 253.3±18.4; Non-exp: 301.8±15.6  - Exp: 254.9±17; Non-exp: 301.8±15.6 |
| Babadi et al. (2021)^27^ (English) | Fencing | Fencer | 28 (Exp: 14, Non-exp: 14) | Dynamic | Participants were asked to only give verbal responses after each video (right fencer-left fencer). | No. of fixations (times) | S of the upper bound  - Exp: 2.17±0.63; Non-exp: 3.03±0.52 |
|  |  |  |  |  |  | Accuracy (%) | S of the upper bound  - Exp: 43.21±4; Non-exp: 27.36±6.4 |
|  |  |  |  |  |  | Mean duration of fixation (s) | S of the upper bound  - Exp: 43.21±4; Non-exp: 27.36±6.4 |
| Gutierrez-Davila et al. (2013)^28^ (English) | Fencing | Fencer | 30 (Exp: 13, Non-exp: 17) | Static | Participants were asked to lunge, attempting to situate the point of the sword inside the circle. | Reaction time (ms) | - Exp: 220±32; Non-exp: 214±26 |
| Martinez de Que et al. (2014)^29^ (English) | Karate | Karate athletes | 21 (Exp: 11, Non-exp: 10) | Dynamic | Participants were required to perform a reverse punch. | Reaction time (ms) | - Exp: 231±51; Non-exp: 271±51 |
| Chung et al. (2012)^30^ (English) | Taekwondo | Taekwondo athletes | 60 (Exp: 20, Mid:20, Non-exp: 20) | Static | Participants responded by pressing the thumb switch and kicking with the dominant leg. | Reaction time (ms) | Sport-specific  - Exp: 296±44.8; Mid: 297±53; Non-exp: 324±45.3 |
| Bianco et al. (2008)^31^ (English) | Boxing | Boxer | 60 (Exp: 27, Non-exp: 33) | Static | Participants were asked to respond to the stimulus by pressing the digit on the computer keyboard. | Reaction time (ms) | Simple reaction time  - Exp: 249±7; Non-exp: 244±7 |
| Del Percio et al. (2007)^32^ (English) | Fencing | Fencer | 23 (Exp: 14, Non-exp: 9) | Static | Participants were requested to respond by pressing buttons with the left or right finger. | Reaction time (ms) | Baseline condition  - Exp: 418±22; Non-exp: 511±27  10-HZ flick condition  - Exp: 420±20; Non-exp: 518±24  15-HZ flick condition  - Exp: 434±22; Non-exp: 537±23 |
|  |  |  |  |  |  | Accuracy (%) | Baseline condition  - Exp: 93.4±2; Non-exp: 95.3±1.7  10-HZ flick condition  - Exp: 92.6±2.6; Non-exp: 94.8±1  15-HZ flick condition  - Exp: 91.9±3.1; Non-exp: 94.8±1.6 |
| Del Percio et al. (2007)^33^ (English) | Karate | Karate athletes | 46 (Exp: 17, Mid: 14, Non-exp: 15) | Static | Participants were requested to respond by pressing buttons with the left or right finger. | Accuracy (%) | Karate attacks  - Exp: 85.4±13.5; Mid: 90.2±14.2;Non-exp: 84.6±12 |
|  |  |  |  |  |  | Reaction time (ms) | Karate attacks  - Exp: 505±115.4; Mid: 619±74.8; Non-exp: 633±201.4 |
| Chen et al. (2016)^34^ (English) | Taekwondo | Taekwondo athletes | 97 (Exp: 38, Non-exp: 35) | Static | Participants were asked to press the left response button according to the target shown on the screen. | Reaction time (ms) | Upper extremity, Dominant side-Valid cue  - Exp: 234±20; Non-exp: 269±29  Upper extremity, Dominant side-Invalid cue  - Exp: 253±19; Non-exp: 291±32  Upper extremity, Nondominant side-Valid cue  - Exp: 232±21; Non-exp: 266±33  Upper extremity, Nondominant side-Invalid cue  - Exp: 249±19; Non-exp: 287±32  Lower extremity, Dominant side-Valid cue  - Exp: 291±30; Non-exp: 326±51  Lower extremity, Dominant side-Invalid cue  - Exp: 383±52; Non-exp: 353±37  Lower extremity, Non-dominant side-Valid cue  - Exp: 293±33; Non-exp: 341±52  Lower extremity, Non-dominant side-Invalid cue  - Exp: 351±34; Non-exp: 387±58 |
|  | Karate | Karate athletes | 97 (Exp: 24, Non-exp: 35) | Static | Participants were asked to press the left response button according to the target shown on the screen. | Reaction time (ms) | Upper extremity, Dominant side-Valid cue  - Exp: 351±34; Non-exp: 387±58  Upper extremity, Dominant side-Invalid cue  - Exp: 276±36; Non-exp: 291±32  Upper extremity, Non-dominant side-Valid cue  - Exp: 252±22; Non-exp: 266±33  Upper extremity, Non-dominant side-Invalid cue  - Exp: 270±33; Non-exp: 287±32  Lower extremity, Dominant side-Valid cue  - Exp: 303±31; Non-exp: 326±51  Lower extremity, Dominant side-Invalid cue  - Exp: 356±45; Non-exp: 383±52  Lower extremity, Non-dominant side-Valid cue  - Exp: 299±33; Non-exp: 341±52  Lower extremity, Non-dominant side-Invalid cue  - Exp: 349±36; Non-exp: 387±58 |
| Williams et al. (2000)^35^ (English) | Fencing | Fencer | 6 (Exp: 3, Non-exp: 3) | Static | Participants were asked to hit the target according to the colored warning light. | Reaction time (ms) | Simple reaction time  - Exp: 340±59; Non-exp: 559±106  CRT2  - Exp: 388±53; Non-exp: 574±98  CRT4  - Exp: 388±75; Non-exp: 575±82 |
| Johne et al. (2021)^36^ (English) | Fencing | Fencer | 60 (Exp: 15, Mid: 15, Non-exp: 15) | Static | Participants lifted a finger from the rest button and pressed the reaction button after noticing the stimulus. | Reaction time (ms) | Simple reaction time for the dominant hand  - Exp: 203.3±10.9; Mid: 233.6±19.8; Non-exp: 253.1± 36.1  Simple reaction time for the non-dominant hand  - Exp: 219.1± 21.7; Mid: 227.3±20.2; Non-exp: 249.2± 30.3 |
| Fontani et al. (2006)^37^ (English) | Karate | Karate athletes | 18 (Exp: 9, Non-exp: 9) | Static | Participants had to press a key when the letter “X” appeared. | Reaction time (ms) | Alert  - Exp: 204±16; Non-exp: 238±31  Alert A  - Exp: 204 ±14; Non-exp: 241±34  Alert B  - Exp: 205±19; Non-exp: 234±30  GNG  - Exp: 476±41; Non-exp: 491±74 |
| Mouelhi Guizani et al. (2006)^38^ (English) | Fencing | Fencer | 24 (Exp: 12, Non-exp: 12) | Static | Participants pressed the button with the thumb of the preferred hand once the stimulus appeared. | Reaction time (ms) | SRT  - Exp: 253.2±27.3; Non-exp: 299.04±40.98  4-CRT  - Exp: 397.2±22.92; Non-exp: 423.73±42.1 |
| Williams et al. (1999)^39^ (English) | Karate | Karate athletes | 16 (Exp: 8, Non-exp: 8) | Dynamic | Participants responded to taped karate offensive sequences presented under anxiety. | Reaction time (ms) | low anxiety  - Exp: 402.8±132.8; Non-exp: 408.2±74.7  high anxiety  - Exp: 297.6±36.1; Non-exp: 360.3±49.2 |
|  |  |  |  |  |  | Accuracy (%) | low anxiety  - Exp: 35.8±6.1; Non-exp: 45.8±5.6  high anxiety  - Exp: 13.8±10.8; Non-exp: 17.5±10.6 |
|  |  |  |  |  |  | Mean duration of fixation(s) | low anxiety  - Exp: 249.7±58.7; Non-exp: 308.5±54.7  high anxiety  - Exp: 328.3±65.2; Non-exp: 288.1±37.2 |
|  |  |  |  |  |  | No. of fixations (times) | low anxiety  - Exp: 4±1; Non-exp: 4.6±1.4  high anxiety  - Exp: 4.3±1.1; Non-exp: 5.7±2.2 |
|  |  |  |  |  |  | No. of fixation places-interest | low anxiety  - Exp: 2.6±1; Non-exp: 2.8±0.9  high anxiety  - Exp: 2.9±1.3; Non-exp: 3.3±1.2 |
| Lesiakowski et al. (2013)^40^ (English) | Boxing | Boxer | 30 (Exp: 15, Non-exp: 15) | Dynamic | Participants were requested to perform a key‐press response to the stimulus constellation. | Reaction time (ms) | - Exp: 850±100; Non-exp: 780±100 |
| Zbigniew et al. (2018)^41^ (English) | Fencing | Fencer | 30 (Exp: 15, Non-exp: 12) | Dynamic | Participants pressed the button with a finger after the bolt hit the palm. | Reaction time (ms) | - Exp: 125.25±24.11; Non-exp: 149.067± 28.255 |

Abbreviations: Exp: Expert group; Mid: Mid-level group; Non-exp: Non-expert group

# Figure S1. Forest plot of accuracy of perceptual anticipation between experts and non-experts


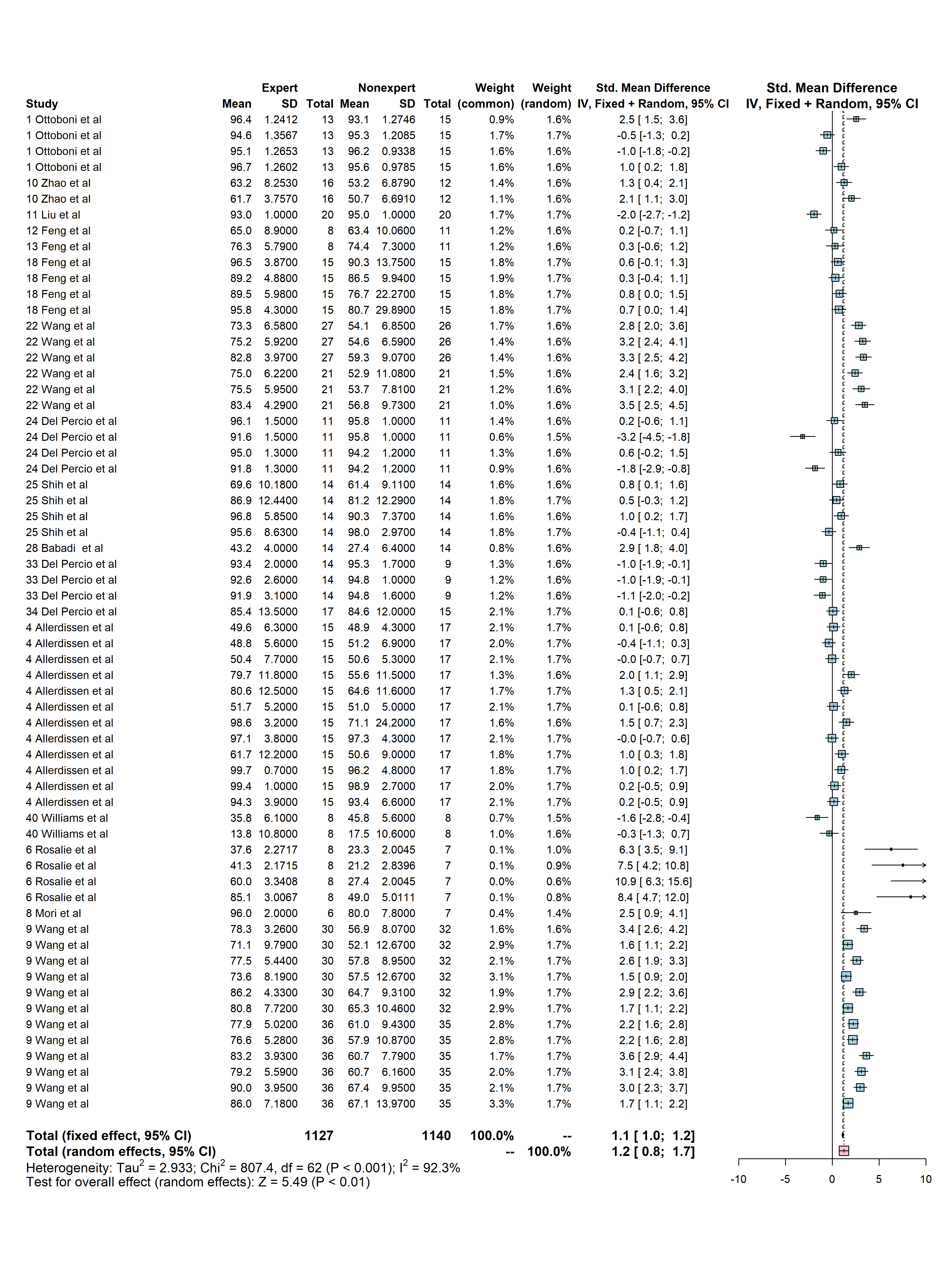


# Figure S2. Forest plot of reaction time of perceptual anticipation between experts and non-experts


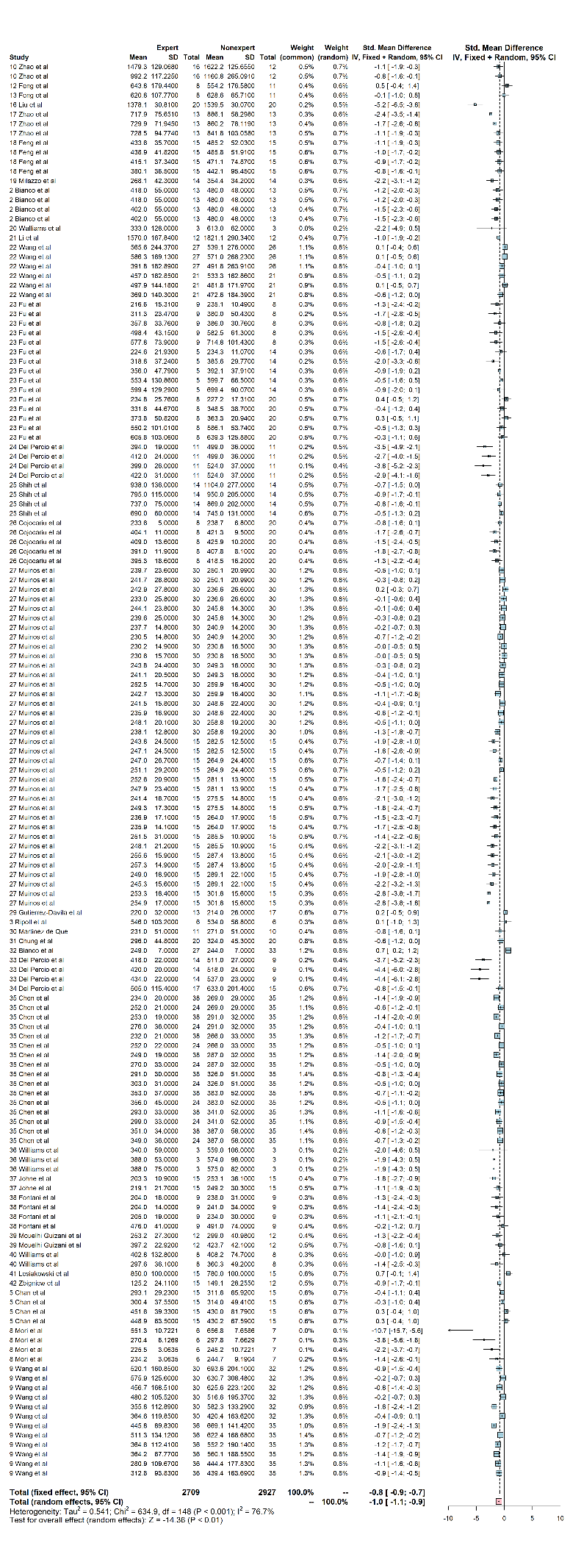


# **References**

1. Ottoboni G, Russo G, Tessari A. What boxing-related stimuli reveal about response behaviour. *Journal of Sports Sciences* 2014; **33**.

2. Bianco V, Di Russo F, Perri RL, Berchicci M. Different proactive and reactive action control in fencers' and boxers' brain. *Neuroscience* 2017; **343**: 260-8.

3. Ripoll H, Kerlirzin Y, Stein J-F, Reine B. Analysis of information processing, decision making, and visual strategies in complex problem solving sport situations. *Human Movement Science* 1995; **14**(3): 325-49.

4. Allerdissen M, Güldenpenning I, Schack T, Bläsing B. Recognizing fencing attacks from auditory and visual information: A comparison between expert fencers and novices. *Psychology of Sport and Exercise* 2017; **31**: 123-30.

5. Chan JSY, Wong ACN, Liu Y, Yu J, Yan JH. Fencing expertise and physical fitness enhance action inhibition. *Psychology of Sport and Exercise* 2011; **12**(5): 509-14.

6. Rosalie SM, Müller S. Timing of in situ visual information pick-up that differentiates expert and near-expert anticipation in a complex motor skill. *Quarterly journal of experimental psychology (2006)* 2013; **66**(10): 1951-62.

7. Piras A, Pierantozzi E, Squatrito S. Visual Search Strategy in Judo Fighters During the Execution of the First Grip. *International Journal of Sports Science & Coaching* 2014; **9**(1): 185-97.

8. Mori S, Ohtani Y, Imanaka K. Reaction time and anticipatory skills of Karate athletes. *Human movement science* 2002; **21**: 213-30.

9. Wang C DM, Chen L. An experimental study on the influence of knowledge representation on intuitionistic thinking decision-making effect of taekwondo athletes under different logic background of representation. *Journal of Beijing Sport University* 2011.

10. Zhao H GC. Study on the Predictive Behavior and Neural Mechanism of Excellent Sanda Athletes under Different Situations. *Journal of Shenyang Institute of Physical Education* 2014.

11. Y F. An ERP study based on the tactical thinking advantage of fencing experts in dynamic situations. *Liaoning Sports Science and Technology* 2015.

12. Y F. An ERP study based on the tactical thinking advantage of fencing experts in static situations. *Journal of Shenyang Institute of Physical Education* 2015.

13. C L. Experimental study on eye movement in Sanda players' visual search. *Journal of Xi 'an Institute of Physical Education* 2010.

14. Y H. Study on eye movement of Sanda players' visual information processing. *Psychological and behavioral research* 2016.

15. Liu S WX. Early processing characteristics of visual search in taekwondo athletes with high anxiety under threat stimulation. *Journal of Southwest Normal University* 2017.

16. H Z. Characteristics and neural mechanism of feature search in elite Sanda players. *Journal of Shanghai Institute of Physical Education* 2010.

17. Feng Y LW, Zhao H, Zhu P. Analysis on the Advantage Characteristics of Orientation Perception Ability of Expert Boxers. *Liaoning Sports Science and Technology* 2018.

18. Milazzo N, Farrow D, Ruffault A, Fournier J. Do karate fighters use situational probability information to improve decision-making performance during on-mat tasks? *Journal of Sports Sciences* 2015; **34**: 1547-56.

19. Williams L, Walmsley A. Response Amendment in Fencing: Differences between Elite and Novice Subjects. *Perceptual and motor skills* 2000; **91**: 131-42.

20. Li A GY. Analysis of visual search advantage of epee fencer in sports situation. *Journal of Wuhan Institute of Physical Education* 2010.

21. C W. Influence of different logic background on intuitionistic thinking effect of taekwondo athletes. *Journal of Beijing Sport University* 2009.

22. C F. Study of characteristics during visual response in high-level fencers. *Journal of the Capital Physical Education College* 2010.

23. Del Percio C, Babiloni C, Infarinato F, et al. Effects of tiredness on visuo-spatial attention processes in elite karate athletes and non-athletes. *Archives italiennes de biologie* 2009; **147**(1-2): 1-10.

24. Shih YL, Lin CY. The relationship between action anticipation and emotion recognition in athletes of open skill sports. *Cognitive processing* 2016; **17**(3): 259-68.

25. Cojocariu A, Abalasei B. Does the reaction time to visual stimuli contribute to performance in judo? *Archives of Budo* 2014; **10**: 73-8.

26. Muiños M, Ballesteros S. Peripheral vision and perceptual asymmetries in young and older martial arts athletes and nonathletes. *Attention, perception & psychophysics* 2014; **76**(8): 2465-76.

27. Babadi Aghakhanpour N, Abdoli B, Farsi A, Moeinirad S. Comparison of Visual Search Behavior and Decision-making Accuracy in Expert and Novice Fencing Referees. *Optometry and Vision Science* 2021; **98**(7).

28. Gutierrez-Davila M, Rojas FJ, Antonio R, Navarro E. Response timing in the lunge and target change in elite versus medium-level fencers. *European journal of sport science* 2013; **13**(4): 364-71.

29. Martinez de Quel O, Bennett SJ. Kinematics of Self-Initiated and Reactive Karate Punches. *Research quarterly for exercise and sport* 2014; **85**(1): 117-23.

30. Chung P, Ng G. Taekwondo training improves the neuromotor excitability and reaction of large and small muscles. *Physical Therapy in Sport* 2012; **13**(3): 163-9.

31. Bianco M, Ferri M, Fabiano C, et al. Comparison of Baseline Neuropsychological Testing in Amateur Versus Professional Boxers. *The Physician and Sportsmedicine* 2008; **36**(1): 95-102.

32. Del Percio C, Brancucci A, Vecchio F, et al. Visual event-related potentials in elite and amateur athletes. *Brain Research Bulletin* 2007; **74**(1): 104-12.

33. Del Percio C, Marzano N, Tilgher S, et al. Pre-stimulus alpha rhythms are correlated with post-stimulus sensorimotor performance in athletes and non-athletes: A high-resolution EEG study. *Clinical Neurophysiology* 2007; **118**(8): 1711-20.

34. Chen W-Y, Wu S, Song T-F, et al. Perceptual and motor performance of combat-sport athletes differs according to specific demands of the discipline. *Perceptual and Motor Skills* 2016; **124**.

35. Williams LRT, Walmsley A. Response timing and muscular coordination in fencing: A comparison of elite and novice fencers. *Journal of Science and Medicine in Sport* 2000; **3**(4): 460-75.

36. Johne M. The impact of fencing training symmetrisation on simple reaction time. *Biomedical Human Kinetics* 2021; **13**(1): 231-6.

37. Fontani G, Lodi L, Felici A, Migliorini S, Corradeschi F. Attention in athletes of high and low experience engaged in different open skill sports. *Percept Mot Skills* 2006; **102**(3): 791-805.

38. Mouelhi Guizani S, Bouzaouach I, Tenenbaum G, Ben Kheder A, Feki Y, Bouaziz M. Simple and choice reaction times under varying levels of physical load in high skilled fencers. *The Journal of sports medicine and physical fitness* 2006; **46**(2): 344-51.

39. Williams AM, Elliott D. Anxiety, Expertise, and Visual Search Strategy in Karate. *Journal of Sport and Exercise Psychology* 1999; **21**(4): 362-75.

40. Lesiakowski. Visuospatial attentional functioning in amateur boxers. *Journal of Combat Sports and Martial Arts* 2013.

41. Zbigniew B. The significance of sensorimotor response components and EMG signals depending on stimuli type in fencing. *Acta Univ Palacki Olomuc* 2008.
